# Genetic assimilation of ancestral plasticity during parallel adaptation to zinc contamination in *Silene uniflora*

**DOI:** 10.1038/s41559-022-01975-w

**Published:** 2023-01-26

**Authors:** Daniel P. Wood, Jon A. Holmberg, Owen G. Osborne, Andrew J. Helmstetter, Luke T. Dunning, Amy R. Ellison, Rhian J. Smith, Jackie Lighten, Alexander S. T. Papadopulos

**Affiliations:** 1 Molecular Ecology and Evolution Bangor, School of Natural Sciences, Bangor University, Environment Centre Wales, Bangor, UK; 2 Fondation pour la Recherche sur la Biodiversité - Centre for the Synthesis and Analysis of Biodiversity, Institut Bouisson Bertrand, Montpellier, France; 3 Ecology and Evolutionary Biology, School of Biosciences, Sheffield, UK; 4 Royal Botanic Gardens, Kew, Richmond, UK; 5 College of Life and Environmental Sciences, University of Exeter, Exeter, UK

**Keywords:** Molecular evolution, Plant evolution

## Abstract

Phenotypic plasticity in ancestral populations is hypothesized to facilitate adaptation, but evidence is piecemeal and often contradictory. Further, whether ancestral plasticity increases the probability of parallel adaptive changes has not been explored. The most general finding is that ancestral responses to a new environment are reversed following adaptation (known as reversion). We investigated the contribution of ancestral plasticity to adaptive evolution of gene expression in two independently evolved lineages of zinc-tolerant *Silene uniflora*. We found that the general pattern of reversion is driven by the absence of a widespread stress response in zinc-adapted plants compared with zinc-sensitive plants. We show that ancestral plasticity that moves expression closer to the optimum value in the new environment influences the evolution of gene expression among genes that are likely to be involved in adaptation and increases the chance that genes are recruited repeatedly during adaptation. However, despite convergence in gene expression levels between independently adapted lineages, ancestral plasticity does not influence how similar expression values of adaptive genes become. Surprisingly, we also observed that ancestral plasticity that increases fitness often becomes genetically determined and fixed, that is, genetically assimilated. These results emphasize the important role of ancestral plasticity in parallel adaptation.

## Abstract (web summary)

Adaptation to new environments often involves changes in gene expression. This study shows a role of ancestral gene expression plasticity in heavy metal adaptation of two independent lineages of *Silene uniflora*.

## Main

The contributions of determinism and contingency in shaping evolution are hotly debated^1–3^. Whether repeated adaptation to the same environment results in similar changes at the molecular level is key to understanding this balance^1,4–6^, as well as the predictability of future responses to environmental change^7^. Adaptation to novel environments often involves gene expression changes, but previous studies have found varying degrees of parallelism during repeated adaptation^8–11^. These changes occur at various levels, including in the overlap of shared differentially expressed genes, fold changes of these genes or final expression levels^9,12^. Understanding the mechanisms that influence the extent of parallelism is an important step in predicting evolutionary responses to new environmental challenges^6,7,13^.

Phenotypic plasticity in ancestral populations (ancestral plasticity) is suspected to play a role in facilitating adaptation to new environments^14–16^. In addition to generally preserving the genetic variability of a colonizing population^17^, plastic responses to new environments could provide the basis for adaptation by moving the trait values in some individuals closer to the new local optimum^18^. Beneficial plasticity of this kind could be retained in locally adapted populations or genetically assimilated and canalized into constitutive expression differences^19^. Alternatively, ancestral plasticity that takes expression levels further away from the new optimum is potentially maladaptive and could hinder adaptation to the novel environment^20,21^.

Current evidence suggests a variety of possible impacts of ancestral plasticity on adaptation^12,21–24^, but the relationship between plasticity and evolutionary parallelism has received limited attention^6,25^. Other properties of gene expression in ancestral populations, such as ancestral expression level or tissue expression location, are associated with increased co-option and potentially parallelism^26,27^. If phenotypic plasticity substantially facilitates the repurposing of traits during adaptation^28^, then beneficial plasticity may result in greater parallelism than when plasticity is maladaptive.

Previous studies have generally found that most ancestral plasticity across transcriptomes is reversed in derived populations, that is, it takes expression values further from the new optimum^22,29–31^ (although there are exceptions^32,33^). However, there are examples of ancestral plasticity in particular genes or traits facilitating subsequent adaptation^18,21,34,35^. Most expression studies on the topic examine transcriptome-wide patterns in ancestrally plastic genes, rarely considering whether genes involved in evolutionary adaptation to the new environment are more likely to have possessed beneficial ancestral plasticity, when compared with the whole transcriptome^20,22,30,31,36,37^. Transcriptome-wide assessments include changes that may not directly contribute to adaptation (in the evolutionary sense), such as those stemming from general stress responses. As a result, estimates of the contribution of ancestral plasticity to adaptation may be distorted in whole transcriptome analysis.

Here we investigate the relationship between ancestral plasticity, adaptation and parallelism using independently evolved lineages of zinc-tolerant *Silene uniflora* from contaminated metal mines and local zinc-sensitive coastal populations^38^. In this species, ancestral coastal populations have repeatedly colonized contaminated mine soils throughout Great Britain and Ireland over the past 250 years^39^, producing locally adapted populations that can grow at high concentrations of zinc^38–40^. As a result, expression differences between closely related mine-coast pairs should resemble the expression differences between the current mine populations and their coastal ancestors. Common changes across replicates are likely to represent adaptive changes rather than drift^6^. Extant coastal populations also provide an approximation of the ancestral plastic response to zinc. This system provides an ideal opportunity to investigate the role of ancestral plasticity in adaptation across multiple evolutionary replicates.

## Results and discussion

**Fig. 1:**
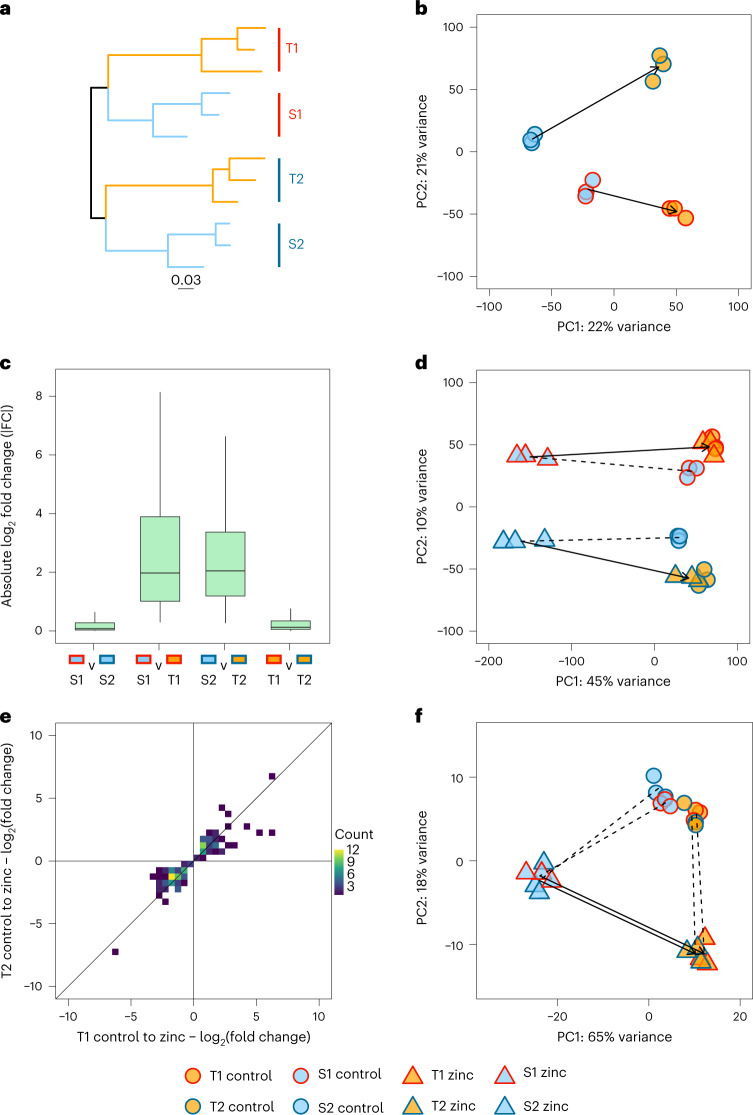
Parallel constitutive and plastic changes in tolerant populations. **a**, Independent origins of the tolerant populations: a maximum-likelihood phylogenetic tree based on 15,285 SNPs; all inter-population relationships had bootstrap support ≥99%. **b**, PCA of variance-stabilizing transformed counts (Methods) of all 27,970 genes for all populations in the control treatment, summarizing constitutive expression differences between populations. Point fill corresponds to zinc tolerance (orange, tolerant; blue, sensitive); point border corresponds to geographic pair (red, Wales (T1/S1); dark blue, England (T2/S2)). Arrows are drawn from the centroid of susceptible populations (S1 and S2) to the centroid of corresponding tolerant populations (T1 and T2, respectively). **c**, For CEC genes, boxplot of absolute values of log_2_-transformed fold changes (|FC|; *y* axis) between pairs of populations (*x* axis) in the control treatment (box, interquartile range; line, median; whiskers, the largest value no further than 1.5× the interquartile range). *N* = 413 for each box. Values above/below whiskers not plotted. **d**, PCA of variance-stabilizing transformed counts in both treatments across all genes. Point fill and border as in **b**. Circles correspond to control treatment, triangles to zinc treatment. Dashed line, plastic change; solid arrow, evolutionary change. **e**, Heat map of log_2_-transformed shrunken fold changes between control and zinc treatments for genes that were differentially expressed between control and zinc in both T1 (*x* axis) and T2 (*y* axis; that is, DP genes). **f**, PCA of variance-transformed counts for DP genes only, in both treatments (legend as in **b**). Source data

Zinc-tolerant populations of *S. uniflora* largely exclude zinc from their shoots, preferentially accumulating zinc in their roots^39,40^. We quantified gene expression in the roots of two independently derived, zinc-tolerant populations from geographically distant, derelict mines (T1, England; T2, Wales) and their nearest and most closely related zinc-sensitive coastal populations (S1 and S2; Fig. 1a). Extant zinc-sensitive coastal populations acted as proxies for ancestral expression. We exposed clones of the same individuals to two treatment conditions (control or zinc-contaminated) and collected RNA-seq data from the roots of the experimental plants. Our experimental design allowed us to quantify: (1) the ancestral plastic response to zinc contamination; (2) the extent of convergent gene expression changes during rapid parallel adaptation; (3) the evolutionary response to ancestral plasticity at a transcriptome-wide level; (4) whether the evolutionary response differs for genes plausibly involved in adaptation; and (5) the relationship between ancestral plasticity and convergent gene expression changes. In so doing, we establish the extent to which rapid adaptation is shaped by constraint and plasticity, disentangling the influence of general stress responses versus adaptive responses on patterns of reversion and reinforcement.

**Extended Data Fig. 1:**
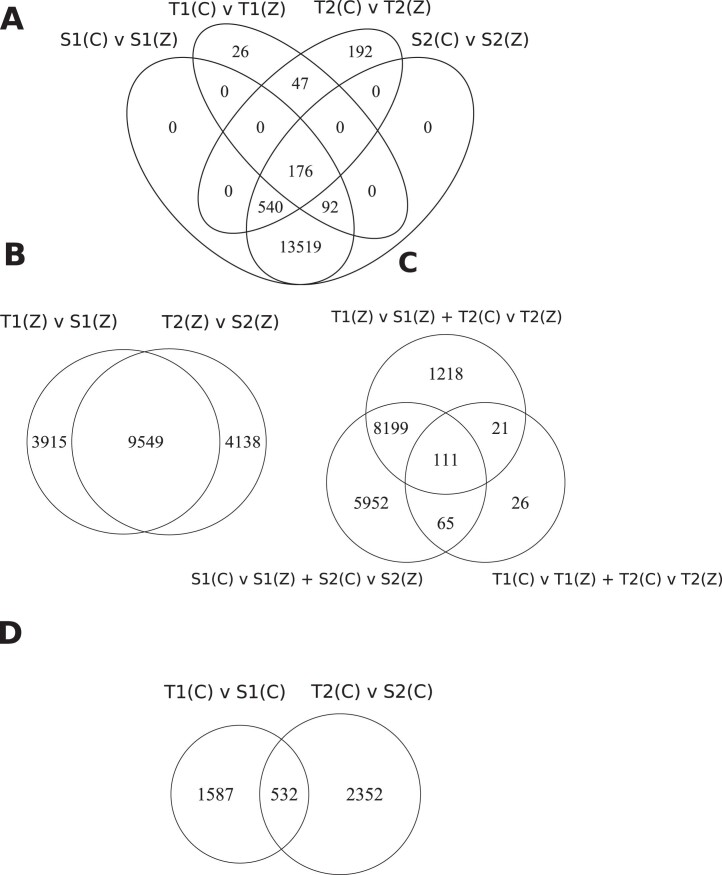
Overlaps between sets of differentially expressed genes. Venn diagrams outlining the numbers of significantly differentially expressed genes for a given comparison (separated by a ‘v’). T1/S1/T2/S2 correspond to populations, (C) corresponds to control conditions, (Z) to zinc conditions: for example, T1(C) v S1(C) refers to genes differentially expressed between T1 in the control, and S1 in the control. A ‘+’ corresponds to genes that fulfil both sets of criteria: for example, T1 (C) v T1 (Z) + T2 (C) v T2 (Z) corresponds to the set of genes that are differentially expressed between both (i) T1 in the control vs. T1 in the zinc, and (ii) T2 in the control vs. T2 in the zinc. Panels illustrate (**A**) between treatment expression changes within populations, and their overlaps, (**B**) between ecotype-changes in the zinc in the two geographic groups, and their overlap, (**C**) the overlap between genes differentially expressed in both tolerant populations between conditions, both susceptible populations between conditions, and between both ecotype pairs within the zinc treatment, and (**D**) between-ecotype changes in the control in the two geographic groups, and their overlap.

**Extended Data Fig. 2:**
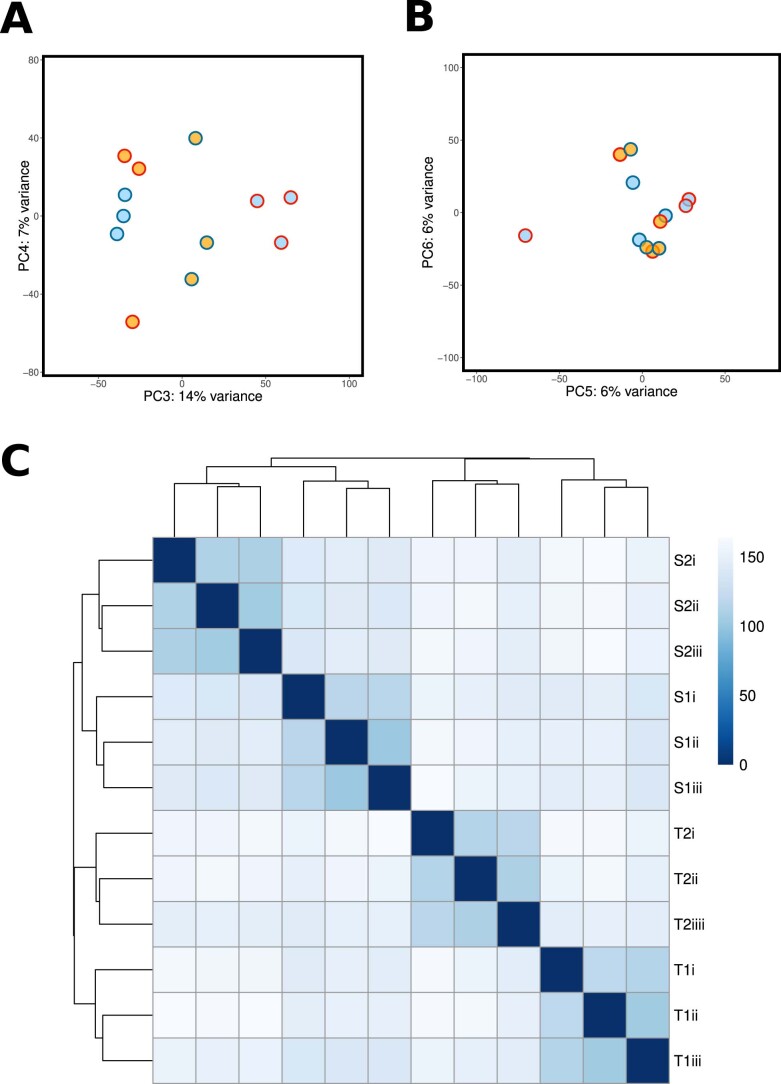
Gene expression variation in control conditions. Within-control variation of counts transformed with a variance-stabilising transform of all genes included in differential expression analysis, for all populations in control treatments, are displayed. A + B) Principal components analysis (PCA), point fill corresponds to population zinc tolerance (orange = tolerant, blue = sensitive), point border corresponds to geographic pair (red = Wales [T1/S1], dark blue = England [T2/S2]). The third and fourth (**A**), and fifth and sixth (**B**) principal components are displayed. (**C**) A heatmap and hierarchical clustering of all individuals in the control treatment based on a matrix of transformed counts. Each individual (i-iii) from each population (S1, S2, T1, T2) is displayed as a row/column. Box colours indicate sample-to-sample distances based on the R dist() function.

Heavy metals are highly phytotoxic and high concentrations of zinc have a considerable impact on growth and fitness of coastal populations of *S. uniflora*^38–40^. Transcriptome-wide ancestral plasticity (that is, the response to zinc in sensitive populations) was dominated by a general and widespread stress response. In total, 51.1% of the transcriptome (14,327 genes) was differentially expressed in both sensitive populations between treatments, with an overwhelming majority being shared across populations (Extended Data Fig. 1a). Shared upregulated genes were enriched for 15 gene ontology (GO) terms related to stress (Supplementary File 1). Further, the major difference in expression between susceptible and tolerant populations was the lack of this extreme response to zinc stress in tolerant populations. Only 223 genes were differentially expressed between treatments in both tolerant populations. In the zinc treatment, 9,549 genes were differentially expressed between tolerant and sensitive populations in both pairs (Extended Data Fig. 1b), which were enriched for 12 stress-related GO terms (Supplementary File 2). Of these genes, 87.0% were ancestrally plastic (that is, also differentially expressed between treatments in both sensitive populations), but only 1.4% showed derived plasticity (DP; that is, were also differentially expressed between treatments in both tolerant populations; Extended Data Fig. 2c). This reveals a substantial disruption to transcription in sensitive plants, consistent with the broad impact of zinc toxicity on cellular processes^41^. It also indicates that, in general, greater transcriptomic perturbations in ancestral populations exposed to new environments may be driven by general stress responses^20,30,36,37^.

### Rapid evolution of highly parallel gene expression changes

**Extended Data Fig. 3:**
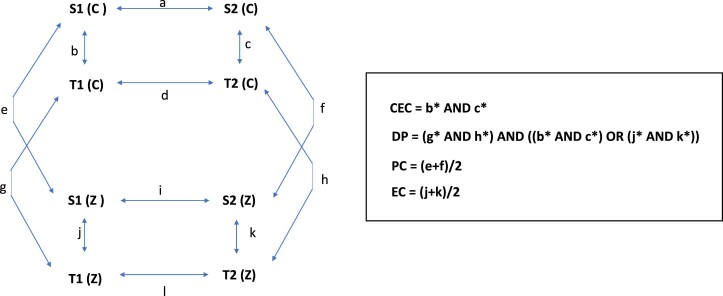
Explanatory diagram. Diagram outlining expression contrasts between populations and treatments, and calculated metrics. Values in bold represent expression values for populations (for example, S1) and treatments (C for control, Z for zinc). Arrows with letter designations represent differences between these expression values (for example, b is the difference in expression levels for a gene in S1 and T1 in the control). The formulae outline the conditions for genes being classified as (i) CEC and (ii) DP, and how (iii) PC and (iv) EC are calculated for individual genes. In the formulae, an asterix denotes a statistically significant difference.

*Silene uniflora* has independently colonized mines and evolved tolerance to the very high levels of zinc (2,400–48,100 ppm) in the contaminated soils^38–40^. Given that this phenotype has evolved in parallel due to strong selection, we also expected a component of the transcription profiles to show parallel changes in tolerant populations. In the control treatment, principal component analysis (PCA) of transcriptome-wide gene expression levels separated populations by zinc tolerance (that is, tolerant versus sensitive) on PC1 and by geographic origin (that is, T1 and S1 versus T2 and S2) on PC2 (Fig. 1b and Extended Data Fig. 3a,b). Within-population variation was low relative to between populations/treatments (Extended Data Fig. 3c). In these benign conditions, the trajectories of whole transcriptome evolution were divergent and almost orthogonal, rather than parallel (sensu ref. ^6^).

**Extended Data Fig. 4:**
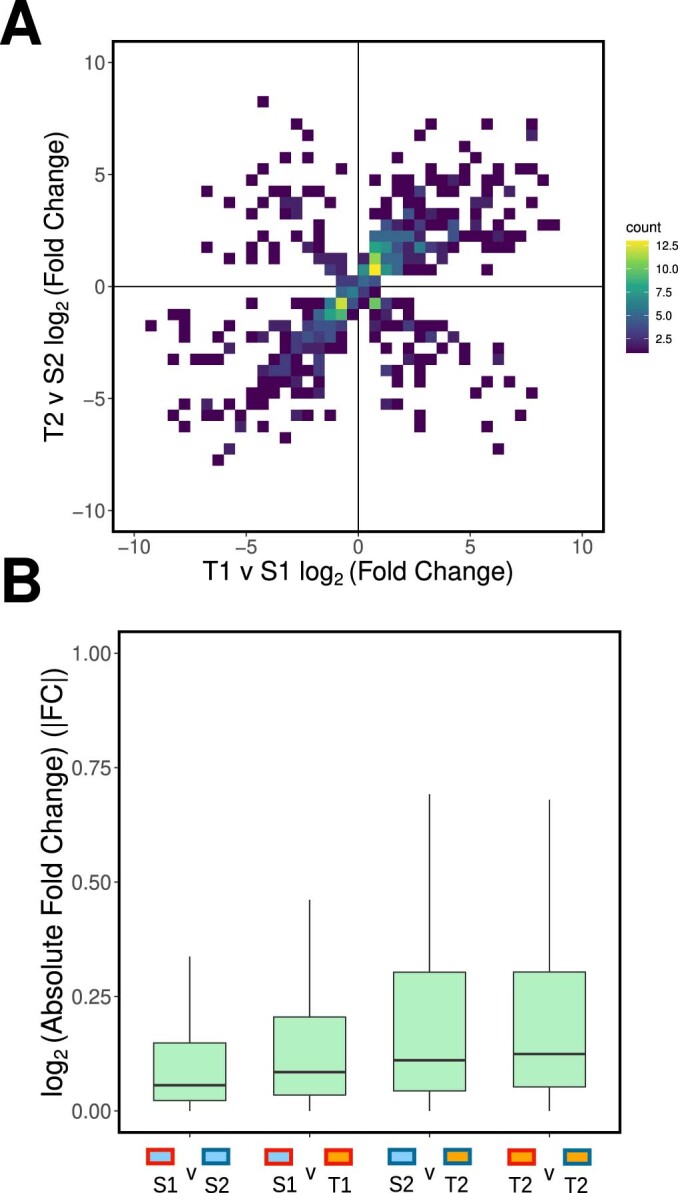
Gene expression similarity in control conditions. (**A**) Heatmap of log_2_ transformed shrunken fold changes between T1 and S1 (x axis) and T2 and S2 (y axis) for genes that are significantly differentially expressed between both T1 and S1, and T2 and S2, in the control treatment. (**B**) Boxplots of absolute values of log_2_ transformed fold changes (|FC|; y axis) between pairs of populations outlined on the x axis, in the control treatment, for all 27,970 genes used in differential expression analysis. Box encompasses 25th to 75th percentiles, line corresponds to median. Whiskers correspond to the largest value no further than 1.5x the interquartile range from either the 25th or 75th percentiles. N = 27,970 for all boxes. Points beyond the whiskers not shown.

In total, 2,119 and 2,884 genes were differentially expressed in control conditions between T1 and S1, and T2 and S2, respectively, of which 532 were shared (Extended Data Fig. 2d). We categorized 400 of these shared genes as displaying parallel constitutive evolutionary changes of expression (CEC genes); these were differentially expressed in both tolerant-sensitive pairs ‘and’ had expression differences in the same direction (that is, increased or decreased expression in both T1 versus S1 and T2 versus S2; Extended Data Figs. 3 and 4a). Genes with expression shifts in the same direction are more likely to be the result of parallel adaptation across the mines^8–11^ and 400 genes represents a greater overlap than expected by chance (one-sided Fisher’s Exact test, odds ratio = 2.2, *P* < 2.2 × 10^−16^). The degree of similarity in gene expression levels between populations can be quantified by comparing the absolute per-gene log_2_-transformed shrunken fold changes (FC; see Methods for rationale). For a set of genes, a small median |FC| indicates high expression similarity between a pair of populations. In control conditions, transcriptome-wide expression levels of tolerant populations were less similar than the coastal populations were to each other (|FC|_S1-S2_ = 0.056 versus |FC|_T1-T2_ = 0.12; two-sided paired Wilcoxon signed-rank test; *V* = 1.2 × 10^8^; *P* < 2.2 × 10^−16^; Extended Data Fig. 4b). The CEC genes had similar expression values in sensitive populations (CEC|FC|_S1-S2_ = 0.077), but expression was also highly similar in tolerant populations (CEC|FC|_T1-T2_ = 0.12), despite substantial expression divergence and genome-wide genetic differentiation from the nearest coastal populations (Fig. 1c; mean *F*_ST_ = 0.36 between susceptible and tolerant populations^38^). In other words, for the 400 CEC genes, parallel evolution in mine populations produced expression similarity comparable to that observed between sensitive populations, which is the product of shared ancestry, gene flow, drift and selection.

**Extended Data Fig. 5:**
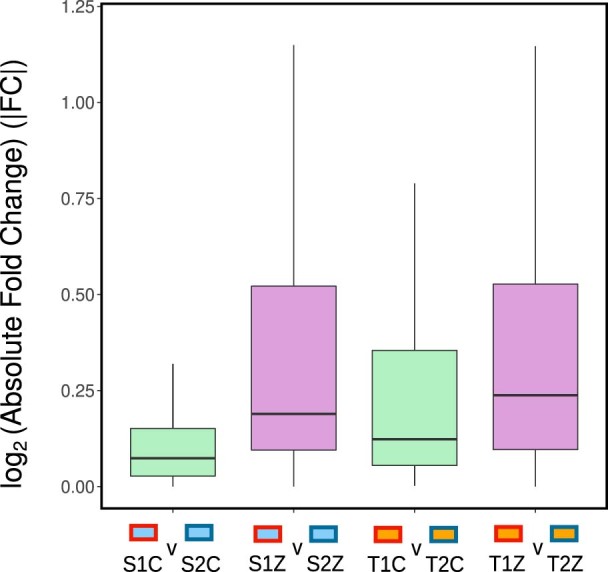
Convergent gene expression in derived plasticity genes. Boxplots of absolute values of log_2_ transformed fold changes (|FC|; y axis) between pairs of populations (x axis) for adaptive plasticity genes, either in the control (green) or zinc (purple) conditions. Box encompasses 25th to 75th percentiles, line corresponds to median. Whiskers correspond to the largest value no further than 1.5x the interquartile range from either the 25th or 75th percentiles. N = 137 for each boxplot. Points beyond the whiskers not shown.

Unlike in the control treatment, there was a higher degree of parallelism in the evolved response to zinc treatment across the whole transcriptome (solid black arrows, Fig. 1d). However, this was largely driven by the widespread transcriptomic response of the sensitive plants, with a less dramatic shift in expression of tolerant populations in zinc versus control treatments. Genes with significant expression responses to zinc in both tolerant populations and that, in both tolerant populations, show expression differences from susceptible populations in either the control or the zinc treatment (or both; Extended Data Fig. 3), are likely to play some role in zinc tolerance (hereafter called DP genes). Of the 245 and 653 genes with this expression pattern in T1 and T2, respectively, 137 were shared. This is a greater overlap than expected by chance (one-sided Fisher’s Exact Test, odds ratio = 66.8, *P* < 2.2 × 10^−16^). This level of parallelism is high compared with other systems, such as repeated adaptation to elevation^42,43^. This difference may be due to the strength and specificity of selection that metal toxicity imposes, rather than more multifarious selection along elevation gradients. These shared genes had highly correlated expression shifts (log_2_ fold changes between treatments; linear model slope = 0.84, *t* = 26, *P* < 2.2 x−10^−16^, adjusted *R*^2^ = 0.83; Fig. 1e). Many DP genes (83%) were also differentially expressed between treatments in both susceptible populations and may constitute a stress response that is partially inherited from their coastal ancestors; indeed, ‘response to stress’ was the most highly enriched GO term for DP genes with ancestral plasticity (Supplementary Files 3 and 4). Nevertheless, there were also convergent changes in expression levels in these genes between tolerant populations. Expression profiles for DP genes were similar in the control treatment, but when exposed to zinc, evolved responses were almost perfectly parallel in tolerant populations (Fig. 1f and Extended Data Fig. 5), consistent with previous studies indicating that phenotypic plasticity can result in increased phenotypic parallelism^25^.

There were three times as many genes with constitutive differences between the sensitive ecotype and the tolerant ecotype (CEC genes) as genes with DP. In the literature, there is considerable variability across taxa in the ratios of constitutive to plastic differences associated with local adaptation^19,30,31,44–48^. This may be a function of the degree to which a stressor varies in strength temporally and spatially within a habitat^39,49,50^. However, CEC genes that do not respond to zinc could be involved in zinc tolerance and/or adaptation to other aspects of the mine environment (for example, exposure, water availability and so on), which may also explain this difference. Overall, these results suggest that highly parallel patterns of differential gene expression across evolutionary replicates can be acquired very early in adaptation and over very short timescales. This is true for both the identity of the genes and the magnitude of expression shifts. Papadopulos et al.^38^ identified both shared and non-shared genetic changes across mine-adapted populations and concluded that there may be a highly polygenic basis to adaptation. These evolved expression shifts could be caused by the same or different underlying genetic variants. The responsible variants may be *cis*- or *trans*-acting, may have arisen via gene duplications, and may either directly affect gene expression or target a few upstream regulators—we are unable to assess this from transcriptomic data alone. Regardless of the nature of the genetic changes that have occurred, they have produced remarkably similar gene expression across independent mine colonization events. Previous experimental evolution studies in *Drosophila*, *Tribolium* and *Ipomoea* have demonstrated the evolution of gene expression plasticity in response to heterogeneous environments within 22–130 generations^20,33,36,51^. We demonstrate that this can also occur in wild plant populations in comparable timeframes and is repeatable between independent colonizations of a novel habitat.

### Convergent zinc tolerance pathways

Examining sets of shared genes with expression patterns consistent with a role in adaptation sheds light on the mechanisms underlying zinc tolerance. CEC genes were enriched for 222 GO terms, including terms associated with metal tolerance (for example, zinc ion transport; see Supplementary File 5). This included homologues of *A. thaliana Zinc Transporter 1* *ZIP1*, which encodes a protein that mediates the uptake of zinc from the rhizosphere^52^, *Heavy Metal Atpase 2* (*HMA2*, encoding a plasma membrane protein that transports zinc from cells^53,54^) and *Metal Tolerance Protein 1* (*ZAT*, encoding a protein that sequesters zinc into vacuoles and controls zinc accumulation in roots^55,56^). These are upregulated in zinc hyperaccumulators such as *Arabidopsis halleri*^57^ and when overexpressed confer increased metal accumulation and tolerance^55,58,59^. The function of these genes is consistent with increased zinc accumulation in the roots of zinc-tolerant *S. uniflora* populations^38,40^. DP genes were enriched for 248 GO terms, including 7 associated with metal tolerance (Supplementary File 6). Two genes are homologues to genes for *A. thaliana* glutathione-s-transferases (GSTs, which have an important role in xenobiotic detoxification^60^). Overexpression of GSTs results in enhanced zinc and cadmium tolerance^61,62^. These GSTs were also differentially expressed between conditions in susceptible populations, further hinting at a role of ancestral plasticity in adaptation. These results indicate that genes that have been repeatedly recruited for a role in zinc tolerance across multiple species^41^ have also undergone repeated gene expression changes in zinc-tolerant populations over a few hundred generations.

### Ancestral plasticity is generally reversed during adaptation

**Fig. 2:**
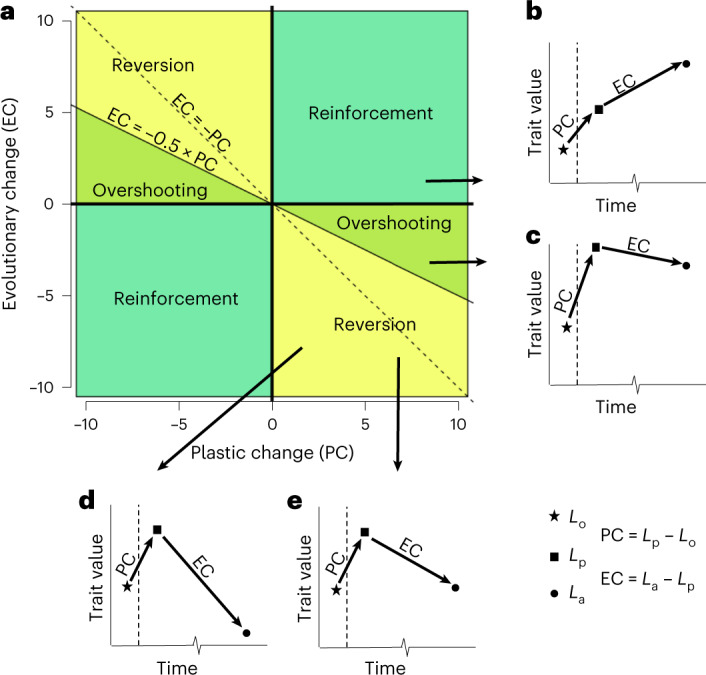
Conceptual overview of evolutionary responses to ancestral plasticity. When an ancestral population reaches a novel environment, an immediate PC moves the trait from an initial value of *L*_o_ in the old environment to *L*_p_ in the new environment. As populations adapt over time, a further EC shifts *L*_p_ to a new value of *L*_a_. **a**, The evolutionary response to ancestral plasticity can be divided into three categories depending on the values of PC and EC. **b**–**e**, Cartoon representations of scenarios. Dashed line represents transition from ancestral to novel environment and associated trait shift, PC. **b**, Reinforcement occurs when the subsequent EC is in the same direction as PC. **c**, Overshooting occurs when PC has moved the trait value closer to the new optimum (that is, *L*_a_ is closer to *L*_p_ than *L*_o_). In this scenario, EC is in the opposite direction to PC, but |EC| < 0.5 × |PC|. **d**,**e**, Reversion occurs when the optimum in the new habitat is nearer to the value of the unstressed ancestor in its home environment than the ancestor’s response (that is, *L*_a_ is closer to *L*_o_ than *L*_p_), so EC is in the opposite direction to PC, but |EC| < 0.5 × |PC|. Reversion can include the restoration of the ancestral state in the old environment (|EC| = |PC|) (**e**) or move beyond this value in the opposite direction (|EC| >|PC|) (**d**). Reinforcement and overshooting suggest that ancestral plasticity was adaptive, whereas reversion indicates it was maladaptive.

To understand the relationship between ancestral plasticity and adaptation, an established approach is to investigate mean differences in gene expression between ancestral populations in their home/control environment (*L*_o_), in a new environment (*L*_p_) and in adapted populations in the new environment^12,22,29,31^ (*L*_a_; see Fig. 2b–e). To make inferences about the role of ancestral plasticity during adaptation, we can compare the direction and magnitude of the initial plastic response of an ancestral population when it is exposed to a new environment (ancestral plasticity/plastic change, PC = *L*_p_−*L*_o_) with the subsequent change in expression between the ancestral population and an adapted population in the new environment^12,22^ (evolutionary change, EC = *L*_a_−*L*_p_). The relationship between PC and EC (that is, the evolutionary response to ancestral plasticity) can be characterized in three ways: (1) ‘reinforcement’, where the initial PC and subsequent EC both move expression in the same direction towards the new optimum (Fig. 2a,b); (2) ‘overshooting’, where PC takes expression beyond the new optimum and EC then adjusts expression in the opposite direction (Fig. 2a,c); and (3) ‘reversions’, where the new optimum is closer to the level of the ancestor in its home environment, so EC largely counteracts the change observed in PC (Fig. 2a,d,e). During both reinforcement and overshooting, the ancestral PC moves expression closer to the new optimum, so both can be interpreted as ancestral plasticity facilitating adaptation to the new environment. Conversely, reversions are likely to be the outcome when ancestral plasticity is maladaptive.

**Fig. 3:**
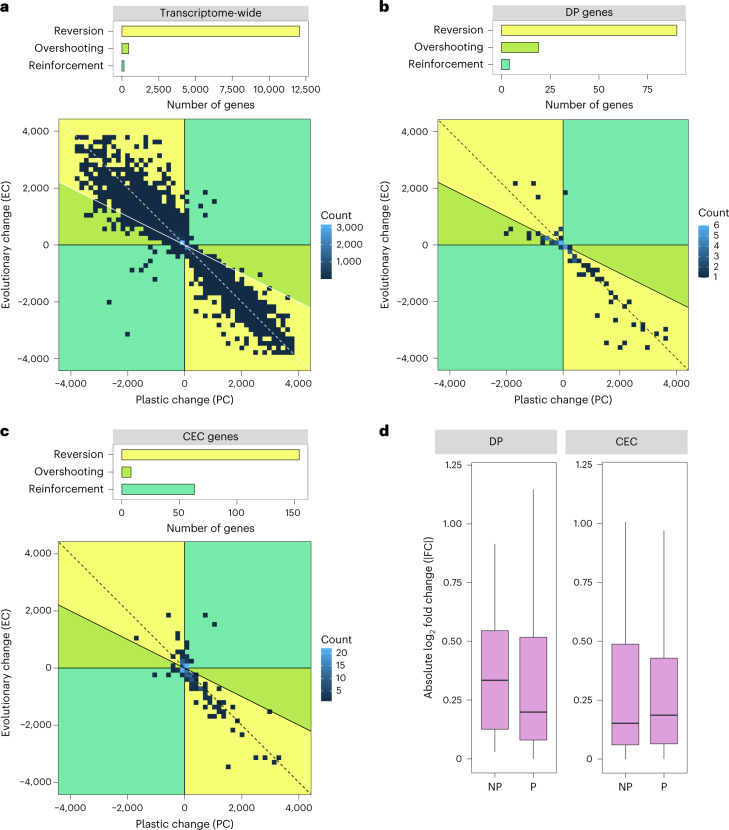
Impact of ancestral plasticity on adaptive evolution and expression convergence. **a**–**c**, Impact on the entire transcriptome (**a**), DP genes (**b**) and CEC genes (**c**). Top: barplots showing numbers of genes displaying reversion, overshooting and reinforcement. Bottom: heat maps of PC versus EC for each gene. Plots display at least 50% of the genes in each category (see Extended Data Fig. 6 for plots of entire datasets). **d**, Boxplots of absolute values of log_2_-transformed fold changes (|FC|; *y* axis) between tolerant populations in zinc for DP genes or CEC genes, and either no ancestral plasticity (NP) or substantial ancestral plasticity (P). Box encompasses 25th to 75th percentiles, line corresponds to median, whiskers correspond to the largest value no further than 1.5× the interquartile range from either the 25th or 75th percentiles. *N*_DP-NP_ = 18, *N*_DP-P_ = 113, *N*_CEC-NP_ = 125, *N*_CEC-P_ = 225. Points beyond the whiskers not shown. Source data

**Extended Data Fig. 6:**
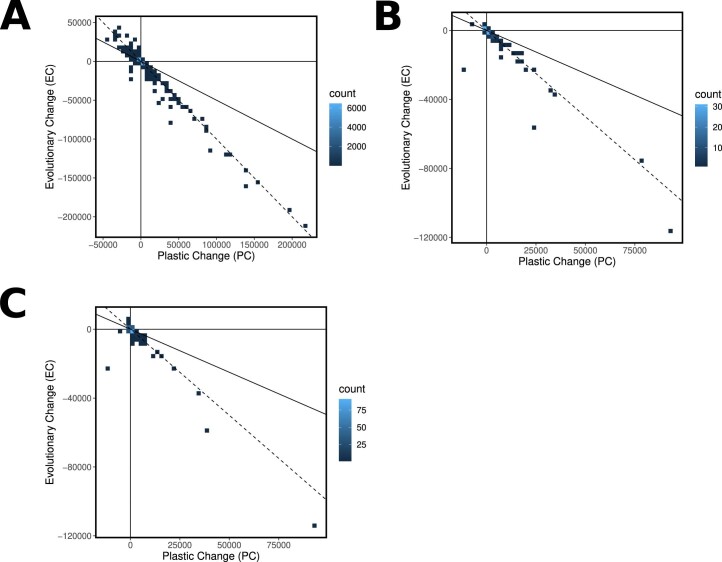
Relationship between PC and EC for different gene sets. Plastic change (PC) vs. evolutionary change (EC) for: (**A**) each gene in the entire transcriptome; (**B**) derived plasticity (DP) genes; and (**C**) genes with constitutive expression differences between ecotypes (CEC).

We evaluated the degree of reversion, reinforcement and overshooting in our transcriptome dataset. To avoid spurious assignment to these categories resulting from very small expression changes, only genes showing substantial changes in PC and EC (those that were differentially expressed: (1) in susceptible plants between conditions (PC); and (2) between mine and coast plants in zinc (EC); see Methods) were placed into these three categories (12,679 genes in total; Fig. 2a). To establish the general pattern of evolutionary responses to ancestral plasticity, we first considered these patterns transcriptome-wide. Across the entire transcriptome, 95.2% of genes showed reversion, with only 1.1% showing reinforcement and 3.7% showing overshooting. Therefore, in the vast majority of cases, ancestral plasticity does not move expression closer to the new optimum (Fig. 3a and Extended Data Fig. 6a). Our transcriptome-wide results are consistent with previous studies in animals and microorganisms, which generally find that reversion is dominant^22,30,36^.

The majority of genes displaying substantial PC and EC across the transcriptome undergo high stress responses in sensitive plants in zinc and remain at unstressed levels in tolerant populations in the zinc treatment. As such, most of the transcriptome is not directly involved in adaptation. Examining the evolutionary response to ancestral plasticity across the predominantly non-adaptive transcriptome provides an indication of the probability that an ancestral plastic response moves expression closer to the new optimum in the zinc-contaminated environment. The large number of subsequent evolutionary reversions indicates that this probability is low (Fig. 3a). Whether this probability increases for genes directly involved in adaptation is more informative for understanding the role of plasticity in adaptation. The DP and CEC genes plausibly have a role in repeated adaptation to zinc as they are consistently recruited across parallel replicates^8–11^, but they account for only 1.8% of the transcriptome. DP and CEC genes are unlikely to include all genes that are involved in adaptation to zinc contamination—some may only be important in a single population or not detected under the framework applied here. Nevertheless, the DP and CEC sets are likely to be enriched for genes involved in adaptation, making them informative as to whether adaptive genes have different responses to ancestral plasticity versus the largely non-adaptive background transcriptomic response.

### Ancestral plasticity is less likely to be reversed in adaptive genes

To understand whether ancestral plasticity facilitates adaptive evolution, we considered the proportion of genes undergoing reversion, reinforcement and overshooting in the DP and CEC gene sets. Among DP genes with substantial PC and EC (82.5% of the total), 79.6% underwent reversion, 3.5% reinforcement and 16.8% overshooting (Fig. 3b and Extended Data Fig. 6b). The higher proportion of overshooting in DP genes relative to the whole transcriptome (16.8% versus 3.7%; *P* = 2.48 × 10^−7^; binomial two-sided test) suggests that DP genes may carry a fitness cost for being expressed at an inappropriate/inaccurate level for a given concentration of zinc and have fine-tuned the ancestral level of plasticity.

Adaptation to zinc contamination has also produced constitutive gene expression differences between tolerant and sensitive populations in the absence of zinc (CEC genes). Ancestral plasticity may facilitate the evolution of differences by moving expression closer to the new optimum, which could then lead to constitutive adaptive changes^19^. Among CEC genes showing substantial PC and EC (56.2% of the total), only 68.4% show signs of reversion, with 28.0% undergoing reinforcement and 3.6% overshooting (Fig. 3c and Extended Data Fig. 6c). This is significantly higher than in either DP genes (28.0% versus 3.5%, *P* < 2.2 × 10^−16^; binomial two-sided test) or transcriptome-wide (28.0% versus 1.1%, *P* < 2.2 × 10^−16^; binomial two-sided test). We conducted parametric bootstrapping as recommended in ref. ^29^ to reduce bias stemming from the presence of *L*_p_ in calculations of PC and EC (Fig. 2). Bootstrapping (see Methods) generally increased the proportion of reversions and reduced the proportion of overshooting, but substantial enrichment of reinforcement in the CEC genes remained (Supplementary Table 1). The increase in reinforcement among CEC genes suggests that ancestral plastic responses make an important contribution during adaptation and may be genetically assimilated in the process.

Here we define genetic assimilation as: when a trait with an environmentally induced response that increases fitness becomes genetically determined and canalized (that is, there is a loss of plasticity)^15,63–65^. Of the 400 CEC genes, 310 are not zinc-responsive in either tolerant population but display substantial PC; these have been repeatedly canalized. Other definitions of genetic assimilation only include cases where the derived trait value is similar to the ancestral value in the new environment^30,66^. Of the 310 canalized genes, 114 do not display substantial EC, that is, the ancestral response was close to the new optimum. These included *HMA2* and *ZAT* (see Zinc tolerance pathways section). For an additional 69 of these canalized CEC genes, the ancestral response took expression closer to the new optimum (that is, overshooting or reinforcement). Altogether 183 genes have undergone genetic assimilation (46% of CEC genes, 0.7% of the transcriptome), emphasizing the importance of ancestral plasticity during rapid adaptation to new environments.

Other studies have looked for a role for ancestral plasticity in producing constitutive expression differences by establishing a positive correlation between ancestral plasticity (which they define as *L*_p_/*L*_o_) and evolutionary change in control conditions (defined as *L*_c_/*L*_o_, where *L*_c_ is the level of the adapted population in the ancestral environment^20,37^). However, the common denominator of *L*_o_ in both variables would tend to produce a positive correlation^67^, potentially making these results unreliable. Most constitutive differences have been found to have evolutionary changes in the opposite direction to ancestral plasticity (reversion and overshooting were not distinguished)^12^, but whether there was an increase compared with the transcriptome-wide pattern was not assessed. Here, we demonstrated that although most ancestral plasticity is maladaptive, ancestral plasticity that can move expression closer to the new optimum contributes to adaptation.

### Ancestral plasticity is not necessary for substantial gene expression convergence

Given this evidence of ancestral plasticity contributing to adaptation, the question of its importance for parallelism in adaptation arises. Plasticity may also increase the propensity of genes to be repeatedly recruited during adaptation. Unlike the shared CEC genes, which had relatively low rates of reversion (68.4%), genes differentially expressed in the control in only one population pair were more likely to show reversion (74.8% in T1/S1 and 80.5% in T2/S2, Supplementary Table 2). In other words, genes repeatedly recruited during adaptation are more likely to have had ancestral plasticity that moved expression closer to the new optimum, than those that were only recruited in one event.

In addition to affecting gene recruitment, ancestral plasticity may also affect the degree of expression convergence in repeatedly recruited genes. Comparisons of expression levels for DP or CEC genes that had ancestral plasticity versus those without returned no significant differences (Fig. 3d; DP genes, |FC|_NOPLAST_ = 0.33, |FC|_PLAST_ = 0.20, two-sided Wilcoxon signed-rank test, *W* = 815, *P* = 0.18; CEC genes |FC|_NOPLAST_ = 0.15, |FC|_PLAST_ = 0.19, *W* = 1.2 × 10^4^, *P* = 0.86). Genes lacking ancestral plasticity can rapidly evolve plastic responses with comparable expression convergence to ancestrally plastic genes. In summary, ancestral plasticity facilitates the repeated recruitment of genes but does not necessarily lead to greater convergence in expression levels during adaptation.

### Experimental considerations

Despite our modest sample size, we controlled for between-treatment expression variation that might stem from genetic differences between individuals within a population by using clones paired across treatments. Further, within-population relative to between-population variability is very low (Fig. 1b and Extended Data Fig. 2c). We acknowledge that the between-residuals effects among cuttings from the same individual may not be zero, but these are likely to be very small given the common starting conditions and identical genotype. Removing the genotype term that pairs individuals across treatments did not alter the observed patterns (Supplementary Table 5). We also could not directly observe expression in the mine populations’ ancestors, but the very recent colonization from coasts means responses in these extant coastal populations are likely to be very similar to the ancestral plastic response. Although some expression shifts might have taken place in the coastal populations since the mine populations diverged, these differences are unlikely to explain the patterns we observed consistently across the replicated events. Additionally, the design limits, but does not eliminate, maternal effects on expression. As such, it is possible that residual maternal effects might have affected some individual genes; however, this would not account for the patterns in large groups of genes and the relationship between putatively adaptive genes and ancestral plasticity. Finally, our experiment only considers gene expression responses; other forms of gene regulation, or mutational effects besides transcription (for example, coding sequence change) could also be important in zinc tolerance evolution.

## Conclusions

Highly parallel gene expression phenotypes have evolved in *S. uniflora* during the repeated colonization of zinc-contaminated mines, despite the short timescales involved and a lack of gene flow between the tolerant populations^38^. By using coastal relatives to approximate the ancestral state, we show that genes displaying beneficial patterns of ancestral plasticity are overrepresented in these highly parallel gene sets, indicating that ancestral plasticity facilitates repeated adaptation to novel environments. The results of our experiment and others confirm that most ancestral plasticity is non-adaptive^22,31,36^. Nevertheless, the considerable proportion of fixed adaptive differences that co-opt ancestral plastic responses suggests that it is a major force in rapid adaptation. Despite a role for ancestral plasticity in enhancing the recruitment of genes, it does not result in an increased level of phenotypic convergence at the level of gene expression compared with genes showing no significant ancestral plasticity. In other words, ancestral plasticity only facilitates parallel evolution at certain levels of biological organization. Overall, our results indicate that genetic assimilation and modification of ancestral plastic responses play an important role in adaptation to novel environments and may be partially responsible for parallelism in gene expression during local adaptation.

## Methods

### Plant materials and experimental procedure

Populations T1, S1, T2 and S2 correspond to WWA-M, WWA-C, ENG-M and ENG-C in ref. ^38^; seeds were collected as described in that study. Three seeds per population, collected from different mothers, were germinated and cuttings propagated at 10 weeks (see Supplementary Methods for conditions). Cuttings were transferred to six deep water culture tanks containing dilute Hoagland’s solution. Susceptible and tolerant populations grow normally in these benign conditions^38–40^. Cuttings from each individual were included in each tank and there was approximately equal representation of populations per tank. The use of cuttings should reduce any maternal effects from differences in resource allocation to seeds between populations. After 1 week of acclimation, the hydroponic solution was replaced with fresh solution in three tanks (control treatment) and the solution adjusted to 600 µM ZnSO_4_ solution in the remaining three tanks (zinc treatment). Eight days later, roots from each individual cutting were flash frozen in liquid nitrogen and stored at −80 °C. For each individual within a treatment, roots of one cutting per tank (three in total) were pooled, homogenized and RNA extracted using a Qiagen RNeasy plant mini kit (see Supplementary Methods for full experimental and extraction conditions). RNA-seq libraries were sequenced at the Beijing Genomics Institute in Hong Kong on a BGISEQ500 with 100 bp paired-end reads (mean insert size 161 bp), producing 25.1–26.0 M read pairs per sample.

### Transcriptome assembly and transcript quantification

After quality control and trimming of sequencing reads (see Supplementary Methods for details), de novo transcriptome assembly was performed using Trinity v2.10.0^68^ using data from one individual per population per treatment. Completeness was assessed using the Eudicots dataset in BUSCO^69^ v.4.0.5: 75% complete (72.2% single copy, 2.8% duplicated), 8.4% fragmented, 16.6% missing. After filtering (see Supplementary Methods for details), 27,970 genes were retained for downstream analysis. Transcripts were annotated using hmmer^70^ 3.3, blastp and trinotate^71^ v3.2.1 (see Supplementary Methods for details).

### Differential gene expression

Abundance estimates for transcripts were summarized at the gene level using tximport^72^ v1.4.2. Gene expression analysis was performed using DESeq2^73^ v1.26.0. Genes with low counts (<10) across all samples were removed. Variance-stabilizing transformed counts for 27,970 genes across all conditions were calculated and used in downstream analysis. This transformation reduces the dependence of the variance on mean expression values, making it more suitable for visualizing between-sample differences^73,74^. Principal components analysis of these counts for (1) all genes in control conditions (Fig. 1b), (2) all genes across all conditions (Fig. 1c) and (3) for DP genes (Fig. 1f) were calculated using the R prcomp function.

Genes differentially expressed between two populations within a treatment (control or zinc) were identified using DESeq2’s in-built models with a single combined factor for population + condition (adjusted *P* = 0.05). Differentially expressed genes between T1 and S1, and T2 and S2 were identified in (1) control and (2) zinc treatments separately using contrasts (see Supplementary Methods section 5 for more details on models and contrasts used for all sets of differentially expressed genes). CEC genes were defined as those differentially expressed between both T1 and S1 in the control, and T2 and S2 in the control in the same direction (that is, both increasing or decreasing in T1 relative to S1 and T2 relative to S2). For between-treatment, within-population comparisons, a model with terms ‘~ Population + Population:Individual + Population:Condition’ was fitted to account for individual-specific variation, which could be accounted for due to pools of clones from each individual being represented in both treatments. Genes differentially expressed between control and zinc treatment were identified for S1, S2, T1 and T2 using individual contrasts (see Supplementary Methods section 5). DP genes were defined as those differentially expressed between conditions in both T1 and T2 in the same direction (that is, both increasing or both decreasing from control to zinc treatment), and were differentially expressed between tolerant and susceptible populations in the control or zinc (or both). The significance of overlaps between sets of differentially expressed genes was determined using a one-sided Fisher’s Exact test. GO enrichment analysis of gene sets was performed using GOseq^75^ v1.38.0 with a false discovery rate of 0.05.

Quantification of fold changes of genes between populations and/or treatments used empirical Bayes shrinkage, calculated with the lfcShrink() function in DESeq2^76^. Values of |FC| were calculated for each gene as the absolute log_2_ fold change between pairs of population/treatment groups (for example, T1 and T2 in the zinc) for a given set of genes. The sign of the log_2_ fold change depends on the order of comparisons being made (for example, a value of +1 between T1 and T2 is equivalent of −1 between T2 and T1); the absolute value must be taken to meaningfully summarize the difference in expression levels (for example, the mean of −2 and +2 would be lower than that of 0.5 and 0.6). The median was used to summarize the values of |FC| as their distribution is highly skewed. Pairwise Wilcoxon signed-rank tests with Benjamini–Hochberg correction were used to detect significant differences in the distributions of |FC| between different pairs of population/treatment groups.

### Classifying responses to ancestral plasticity

To classify evolutionary responses to ancestral plasticity in the transcriptome-wide, DP and CEC gene sets, the following parameters were calculated for each gene: *L*_o_, mean expression value across S1 and S2 in control; *L*_p_, mean expression value across S1 and S2 in zinc; *L*_a_, mean expression value across T1 and T2 in zinc. These were used to calculate the initial plastic change (PC = *L*_p_−*L*_o_) and subsequent evolutionary change (EC = *L*_a_−*L*_p_) for each gene, as in ref. ^12^. Only genes with substantial plastic and evolutionary change were assigned as undergoing reversion, reinforcement or plasticity (very small values of EC or PC due to measurement error would lead to spurious assignment of genes to categories^22^). Genes were defined as having substantial (1) PC if they were differentially expressed between conditions in susceptible populations, combining data across S1 and S2 (using model ~Ecotype + Ecotype:Individual_plant + Ecotype:Condition, and contrast EcotypeS.CondZ, where ecotype (S1, S2) = S and (T1, T2) = T) and (2) EC if they were differentially expressed between tolerant and susceptible populations in zinc, combining data across both population pairs (using model ~Eco_Cond; a combined term of ecotype and condition, and the contrast SZ versus TZ). Data across ecotypes were combined to gain maximum power to detect small shifts in expression; alternative approaches outlined in Supplementary Methods gave similar results. Genes were assigned to one of three categories of evolutionary response to ancestral plasticity^36^: (1) Reinforcement, if EC×PC > 0; (2) Overshooting, if EC×PC < 0 and |EC | < 0.5×|PC|; or (3) Reversion, if EC×PC < 0 and |EC| > 0.5×|PC|. Significant differences in the relative proportions of these categories between sets of genes (for example, CEC genes compared to the transcriptome as a whole) were assessed using a two-tailed binomial test. Parametric bootstrapping of gene assignment to these categories following ref. ^29^ was implemented in R and repeated 100 times per gene (see Supplementary Methods); classification of genes passing this threshold is reported in Supplementary Table 1. For genes showing DP/CEC expression patterns but in T1/S1 or T2/S2 only, values of *L*_o_, *L*_p_, *L*_a_, EC and PC were only calculated using the samples from T1/S1 and T2/S2 separately (Supplementary Table 2) and categorized on the basis of these values. Assignment of categories for transcriptome-wide, CEC and DP genes was also calculated using T1/S1 and T2/S2 separately; these did not differ substantially between evolutionary replicates or the combined calculations (Supplementary Table 2).

### Genotyping

For genotyping, cleaned reads were mapped to the transcriptome using HISAT2^77^ v2.2.1. Genotypes were called using bcftools and a phylogenetic tree was constructed on the basis of 15,285 single-nucleotide polymorphisms (SNPs) using SNPhylo^78^ v20180901 (see Supplementary Methods for details).

### Reporting summary

Further information on research design is available in the Nature Portfolio Reporting Summary linked to this article.

## Supplementary information

Supplementary Information Supplementary Methods 4, legends for Supplementary Data 1–8, Supplementary Tables 1–5 and Supplementary References.

Reporting Summary

Supplementary Data 1 For genes upregulated from control to zinc in both S1 and S2 in the same direction, table outlining enriched GO terms.

Supplementary Data 2 For genes differentially expressed between T1 and S1, and T2 and S2 in the zinc treatment in the same direction, table outlining enriched GO terms.

Supplementary Data 3 For genes displaying the DP expression pattern (see main text for definition), which also showed differential expression between treatments in S1 and S2, table outlining enriched GO terms.

Supplementary Data 4 For genes displaying the DP expression pattern (see main text for definition), but which did not show differential expression between treatments in S1 and S2, table outlining enriched GO terms.

Supplementary Data 5 For genes displaying the CEC expression pattern (see main text for definition), table outlining enriched GO terms.

Supplementary Data 6 For genes displaying the DP expression pattern (see main text for definition), table outlining enriched GO terms.

Supplementary Data 7 Data for each individual sequenced.

Supplementary Data 8 Phylip format file of sites used to construct the phylogenetic tree in Fig. 1a.

## Data Availability

RNA-seq data are deposited on the NCBI databases under Bioproject PRJNA706929. Source data are provided with this paper.

## Source data

Source Data Figs. 1 and 3, and Extended Data Figs. 1, 2 and 4–6 Matrix of counts for all 27,970 genes used in the differential expression analysis for each sample in the experiment. Produced using the DESeq2 assay() function of the DESeqDataSet object

## Code availability

The R code used to analyse the gene expression data is available at https://github.com/danielwood1992/Silene_RNASeq.
