# Supplementary material for: Genetic assimilation of ancestral plasticity during parallel adaptation to zinc contamination in Silene uniflora
Source: Nat Ecol Evol. 2023 Jan 26;7(3):414–23. doi: 10.1038/s41559-022-01975-w (PMC9998271; doi:10.1038/s41559-022-01975-w)
Supplement: Supplementary file 1 — Supplementary Methods 4, legends for Supplementary Data 1–8, Supplementary Tables 1–5 and Supplementary References. [file 41559_2022_1975_MOESM1_ESM.pdf]

nature ecology & evolution

Article

https://doi.org/10.1038/s41559-022-01975-w

Genetic assimilation of ancestral plasticity during parallel adaptation to zinc contamination in *Silene uniflora*

In the format provided by the authors and unedited

This PDF file includes:

Supplementary Methods
Legends for Supplementary Files 1-7
Supplementary Tables 1-5
Supplementary References

Other supplementary materials for this manuscript include the following:

Supplementary Files 1-8

Supplementary Methods

1. Plant materials and experimental procedure

Seeds from each of the four populations were collected as described in Papadopulos *et al.*[1]. Populations T1, S1, T2 and S2 correspond to populations WWA-M, WWA-C, ENG-M and ENG-C in Papadopulos *et al.*[1]. In October 2019 wild-collected seeds (three seeds per population, collected from different individuals) were germinated in Erin Traditional Multipurpose Compost in 80ml cells and grown in a greenhouse (16/8 hour day/night cycle; temperature controlled 18/12 ºC, supplementary lighting automatically switched on if light levels fell below 120 μmol m^{-2} s^{-1} during the day) before being transplanted to 1.5L pots filled with compost and 14.7g MiracleGro Slow Release Fertiliser after five weeks**.** When the plants were ten weeks old at least six 6-8cm cuttings per plant were taken per plant and suspended in a foam discs (1 disc per individual) in a 40 Site X-Stream Aeroponic Propagator filled with 12L Ultrapure deioinised water (Barnstead D4642-33 e-pure ultra water purification system). After 2.5 weeks the propagator was refreshed with 1.92g Hoaglands No. 2 Basalt Salt Solution (Merck) dissolved in 12L ultrapure water once a week for a further three weeks to encourage root growth. This second round of root development, after being grown from seed in common benign conditions, should reduce any maternal effects from differences in resource allocation to seeds between populations.

The rooted cuttings were then transferred to six hydroponics tanks. For each tank, 1.28g Hoaglands No. 2 Basal Salt Solution was dissolved in 0.8L deionised water (Fluid Science) and adjusted to pH 5.5 using 0.1M Potassium Hydroxide. This was added to an 18L opaque tank (48 x 39 x 20cm) and topped up to 8L using deionised water. Tanks were aerated using an airstone and pump. Individual cuttings in foam discs were added to 5x4

polystyrene floats, with 18-19 cuttings per tank, with cuttings from each individual present at least once in each tank and approximately equal representation of each population in each tank.

After one week of acclimation, the hydroponic solution was replaced. For three of the six tanks, fresh hydroponics solution prepared as previously described was added (control treatment). For the remaining three tanks, 1.28g of Hoaglands No2 Basal Salt solution and 2.4mL 2.0M $ZnSO_4$ solution (Sigma) were dissolved in 0.798 ml deioinised water, and adjusted to pH 5.5 as above. This was then topped up to 8L with deioinised water, giving a final $ZnSO_4$ concentration of 600µM (zinc treatment). Eight days after media replacement, the roots from each individual cutting were flash frozen in liquid nitrogen and stored on dry ice before being transferred to a -80˚C freezer. From each individual within a treatment, one cutting's roots from each of the three tanks (which were of approximately equal size) were pooled. This resulted in each of the three individuals per population being represented by a pooled sample of three cuttings (one from each of the three replicate tanks), for both the control and the zinc treatment. This approach reduced technical variation from between-cutting and between-tank differences, and allowed between-individual variation to be accounted for when comparing within populations between the two treatments.

2. RNA extraction and sequencing

Roots from each sample were homogenised on dry ice using a pestle to evenly distribute material from each of the three replicate cuttings. From this, a volume of approximately 150µL of material was added to 2.0mL Safe-Lock Tube (Eppendorf) along with a 5mm

stainless steel bead (Qiagen). Samples were homogenised using a TissueLyser LT (Qiagen), the adapter of which had been chilled in a -80˚C freezer for at least 2 hours, for 1 minute at 50Hz. RNA was extracted using a Qiagen RNeasy Plant Mini Kit (Qiagen) using the standard protocol, including the optional DNase digestion step, with two 50µL elutions in RNase free water. RNA quantity and quality were checked using electrophoresis on a 1% agarose gel, a Nanodrop v2 and Qubit BR RNA Assay Kit (Thermo Fisher) using a Qubit v3. Samples were shipped on dry ice to the Beijing Genomics Institute in Hong Kong and RNA integrity measured using an Aligent Technologies 2100 Bioanalyser, with RIN ranging from 8.2-10.0 (Supplementary File 7). RNA-seq libraries were prepared according to the BGISEQ-500 RNA-Seq Library Preparation Protocol (Document NO: SOP-SS-027) with mRNA enrichment using oligo(dT)-attached magnetic beads. Libraries were sequenced on a BGISEQ500 with 100bp paired-end reads with an average insert size of 161bp. This produced 25.1-26.0M read pairs per sample (Supplementary File 7).

3. Data pre-processing, transcriptome assembly and transcript quantification.

Read quality was inspected using FastQC[2] v0.11.8. The IlluQC.pl script from NGSQCToolkit[3] v2.3.3 was used to keep reads with at least 70% of the sequence having a PHRED score >20. Trimmomatic[4] v0.39 was used with settings LEADING:10 TRAILING:10 SLIDINGWINDOW:4:15 MINLEN:70 to remove leading and trailing low-quality bases with a length cut-off of 70bp. Trimmed reads were inspected for quality and adapter contamination using FastQC.

De novo transcriptome assembly was performed using Trinity[5] v2.10.0 using -min_kmer_cov=2 and default settings for all other parameters. The reads from control and

zinc from the individual in each population with the highest total number of reads were used to construct the transcriptome (Supplementary File 7). This resulted in a transcriptome of 502,579 transcripts from 270,649 trinity "genes" (henceforth referred to as genes). Transdecoder[5] v.5.5.0 was used to predict open reading frames (ORFs) and transcripts lacking an ORF at least 100 amino acids long, which are likely to mostly be assembly artefacts, were discarded. 237,424 transcripts from 121,009 genes were retained. Reads were mapped to these transcripts using the align_and_estimate_abundance.pl utility in Trinity, using kallisto[6] v0.46.1.

To examine whether short-read transcriptome assembly may present difficulties in accurately reconstructing transcripts, we calculated Ex90N50 using the trinity contig_ExN50_statistic.pl utility, giving a value of 2,316bp. This indicates that the N50 of the most highly expressed 90% of the transcriptome is 2,316. An iso-seq transcriptome, which uses longer PacBio reads for de novo assembly, was recently published from *Silene noctiflora*[7]. This had an N50 value of 1,857, suggesting our transcriptome assembly is of comparable contiguity to one constructed using long-read sequencing technology. Transcriptome completeness was assessed using BUSCO[8] v.4.0.5 using the Eudicot dataset. For these highly conserved single copy genes our transcriptome assembly is 72.2% single copy, 2.8% duplicated, 8.4% fragmented and 16.6% missing. This is a level of completeness comparable to the *S. uniflora* genome assembly [1], indicating a low percentage of BUSCO transcripts being inappropriately fragmented or deleted. These results indicate little inappropriate fragmentation or duplication of genes in our assembly.

Individual de novo assemblies may reduce the chances of genetically divergent homologous transcripts being resolved into separate genes, but reducing the amount of data

per assembly may also reduce the number of transcripts that can be assembled. To evaluate the impact of multiple vs individual assemblies, the individuals used in the joint transcriptome assembly were assembled individually and completeness/duplication assessed using BUSCO. These results (Supplementary Table 4) indicate that a smaller proportion of BUSCOs are assembled in the individual de novo assemblies, and the combined assembly does not contain substantially more duplicated BUSCOs than the individual assemblies. It is possible that polymorphic genes may be assembled into different isoforms within a single gene. However, we use tximport to summarise expression values of all isoforms to the gene level, so even if reads from some individuals map to different isoforms within a gene, this will not affect the expression level of the genes. We therefore conclude that our combined assembly is best placed to accurately identify expression levels of the most genes.

To identify and remove transcripts potentially representing microorganism contaminants from the hydroponic media, for each gene the longest transcript was mapped to the *Silene uniflora* genome (NCBI: ASM1898310v1)[1] using BLAT[9] v35, with the database translated into six frames to protein and query three frames into protein using the arguments -q=RNAX -t=DNAX. Genes with a match >200bp and >90% sequence identity were retained. To avoid discarding genes with homologs in unassembled regions of the *S. uniflora* genome, the translated protein sequences of the transcripts were searched against the SwissProt/UniProt[10] database [version 290; using BLASTP v2.10.0[11]]**.** Genes with a top hit to an Embryophyte with match length >200bp and >70% sequence identity were also retained. This resulted in the retention of 27,970 genes for use in downstream analysis.

To identify how many recently duplicated genes may be collapsed into a single contig in our assembly, we took BLAT matches from our filtered transcriptome that matched the *S. uniflora* genome with >200bp length and >90% sequence ID. So that locus to contig relationships could be unambiguously identified, we chose regions that had a single contig mapped; 940 contigs mapped to these regions. Of these, 86 had a hit on a different scaffold of which 77 had a match length >95% of the length of the first match; these contigs could represent expression products of these multiple loci being collapsed. This suggests that a small proportion of contigs (<10%) may result from gene expression of multiple loci.

Merging multiple genes that have expression differences between conditions may cause those differences to be dampened or cancel each other out, but would not result in increased signal. We do not think that contigs consisting of duplicated genes should be excluded either - changes in gene copy number has been shown as a driver of gene expression change (e.g., Stranger et al. 2007[12]) including in metal tolerance (e.g., Craciun et al. 2012[13]). We would also note that genes with highly similar sequences may be hard to resolve during genome assembly, and unambiguously mapping reads to one vs. another copy of a gene would be comparably challenging to a reference genome assembly as it would be to a de novo transcriptome."

To annotate the transcriptome, ORFs >100 amino acids were searched against the Pfam-A[14] v33.0 database using hmmer[15] v3.3 to identify protein domains. These results along with the blastp results described above were loaded into an SQLite database using trinotate[16] v3.2.1.

4. **Defining genes as having undergone substantial PC and EC**

Only genes having "substantial" PC and EC were investigated for their subsequent response to ancestral plasticity, to avoid spurious classification into these categories for genes with very small expression shifts. There are a number of possible approaches to define this. 1. Ghalambor *et al.*[17] and other studies in the field have used a fixed cutoff of 0.2 x L_o for the magnitude of both EC and PC. However, genes with high expression variability may have average expression values distorted by a single outlier individual. 2. Using a cutoff of 0.2 x L_o for the magnitude of EC and PC, and additionally statistically significant expression shifts for both S1 and S2 between conditions (PC) and S1 and T1, and S2 and T2, in the zinc (EC). This approach results in a much larger proportion of genes being discarded (Supplementary Table 3). Too stringent an approach may risk distorting the results; overshooting in particular is characterised by a relatively small expression shift in EC compared to PC. Expression shifts in adaptive genes (Fig. 3B, 3C) are relatively small and may be particularly sensitive to such an approach. 3. Defining substantial PC as a significant expression shift in both S1 and S2, combining data across both populations (using a model ~Ecotype + Ecotype:Individual_plant + Ecotype:Condition), and defining EC as a significant expression shift from susceptible to tolerant individuals in the zinc (using a model with a single combined term for Ecotype and Condition). This approach increases the power to detect small shifts in expression by combining replicates whilst avoiding the problem of highly variable genes distorting the mean. Whilst this approach may be problematic for genes with different behaviours between geographic pairs, the CEC and DP gene sets were defined as those showing highly similar behaviours across both populations pairs. The third method was used in the main text; all three approaches are

compared in Supplementary Table 3. The second method gives a relatively high proportions of genes undergoing reinforcement and overshooting; whereas the third gives many more genes undergoing reversion in all comparisons, with the first method giving an intermediate number of reinforcements and overshooting. All three methods show a substantial enrichment in genes undergoing reversion in CEC genes, and a smaller enrichment of genes undergoing overshooting in DP genes.

5. Parametric bootstrapping for classifying responses to ancestral plasticity

Parametric bootstrapping was implemented in R following the method of Ho & Zhang[18]; for each gene normal distributions were generated with means L_o, L_p and L_a and sd the standard error of L_o, L_p and L_a as estimated from the data (i.e. from across the 6 individuals used to generate each parameter). Values were randomly drawn from each distribution and from these the gene was classified as reversal, overshooting or reinforcement. This was repeated 100 times per gene and genes which did not show one classification in 95 or more repeats were excluded from the analysis. The results of this analysis for the entire transcriptome, CEC and DP genes are shown in Supplementary Table 1.

4. Genotyping and phylogenetic inference

For genotyping, cleaned reads from each individual were mapped to the longest isoforms of each Trinity gene using HISAT2[19] v2.2.1, with reads with MAPQ <20 removed using samtools[20] v1.7. Genotypes were called using bcftools v1.10.2[21]. Indels were removed, and at least 2 genotyped individuals per population were required for a site to be included, with a minimum read depth of 6 for a sample to be genotyped. Per-population vcfs were merged

and sites with QUAL<20 removed. Only sites in the filtered genes used in the gene expression analysis were included. A phylogenetic tree was constructed using SNPhylo[22] v20180901 using default settings, which removes sites with linkage disequilibrium >0.1, missing rate >0.1 and minor allele frequency <0.1, and constructs a maximum-likelihood tree. This resulted in a tree generated from 15,285 SNPs, with 100 non-parametric bootstrap replicates performed to assess branch support. All inter-population branches had bootstrap support >= 99%. (Figure 1A).

5. Models and contrasts used for differential gene expression

To identify sets of differentially expressed genes, models were fit to the entire dataset in DESeq2 using the ~design() function. Contrasts were then used to identify sets of differentially expressed genes within subsets of the dataset using the results() function. Sections 5.1-5.4 show levels of each factor assigned to each individual, how these were combined into a model, and the subsequent contrasts used to define sets of differentially expressed genes. Subsets, unions or intersections of these genes form the CEC, DP and other sets of genes described in the main text.

To identify genes differentially expressed between populations within each condition, a model with a single factor representing all the combinations of population and condition was fitted (Pop_Cond) and the relevant contrasts identified as recommended in the Interactions section of the DESeq2 user guide (Section 5.1 below). For between-conditions comparisons, the paired pools of clones between conditions (i.e. individual i1 of population S1 was present in both C and Z) allowed us to fit a model that accounted for differences due to individual genotypes (Section 5.2) below, as specified in the "group-specific condition effects, individuals nested within groups" section of the DESeq2 user guide.

Analagous models were fit, using an ecotype term (S vs. T) rather than population (S1, S2, T1, T2) to identify genes with substantial |PC| and |EC| (see Section 4 above for justification; Sections 5.3 and 5.4 below for models and contrasts used). The impact of not including a genotype specific term was assessed by identifying between-condition changes using contrasts from the ~Pop_Cond and ~Eco_Cond models, using the contrasts T1C vs. T1Z, T2C vs. T2|, S1C vs. S1Z, S2C vs. S2Z at the population level and TC vs.TZ, SC vs. SZ at the ecotype level respectively, which do not include the effect of genotype. The results of this are displayed in Supplementary Table 5; fewer genes with between-condition changes are identified, but this has minimal impact on our estimates of evolutionary responses to ancestral plasticity across different gene sets.

5.1 Within-condition comparisons (population level):

Individual assignments:

Sample	Pop_Cond
T1_i1_C	T1C
T1_i1_Z	T1Z
T1_i2_C	T1C
T1_i2_Z	T1Z
T1_i3_C	T1C
T1_i3_Z	T1Z
S1_i1_C	S1C
S1_i1_Z	S1Z
S1_i2_C	S1C
S1_i2_Z	S1Z
S1_i3_C	S1C
S1_i3_Z	S1Z
T2_i1_C	T2C
T2_i1_Z	T2Z
T2_i2_C	T2C
T2_i2_Z	T2Z
T2_i3_C	T2C
T2_i3_Z	T2Z
S2_i1_C	S2C

S2_i1_Z	S2Z
S2_i2_C	S2C
S2_i2_Z	S2Z
S2_i3_C	S2C
S2_i3_Z	S2Z

Design matrix:

~Pop_Cond

Contrasts:

**Contrast**	**Genes**	**Gene set**
S1C vs. S2C	S1 vs. S2 in control	a (Extended Data Fig. 3)
S1C vs. T1C	S1 vs. T1 in control	b (Extended Data Fig. 3)
S2C vs. T2C	S2 vs. T2 in control	c (Extended Data Fig. 3)
T1C vs. T2C	T1 vs. T2 in control	d (Extended Data Fig. 3)
S1Z vs. S2Z	S1 vs. S2 in zinc	i (Extended Data Fig. 3)
S1Z vs. T1Z	S1 vs. T1 in zinc	j (Extended Data Fig. 3)
S2Z vs. T2Z	S2 vs. T2 in zinc	k (Extended Data Fig. 3)
T1Z vs. T2Z	T1 vs. T2 in zinc	l (Extended Data Fig. 3)

5.2 Between-condition comparisons (population level)

Individual assignments:

**Sample**	**Pop**	**Cond**	**Ind**
T1_i1_C	T1	C	1
T1_i1_Z	T1	Z	1
T1_i2_C	T1	C	2
T1_i2_Z	T1	Z	2
T1_i3_C	T1	C	3
T1_i3_Z	T1	Z	3
S1_i1_C	S1	C	1
S1_i1_Z	S1	Z	1
S1_i2_C	S1	C	2
S1_i2_Z	S1	Z	2
S1_i3_C	S1	C	3
S1_i3_Z	S1	Z	3
T2_i1_C	T2	C	1
T2_i1_Z	T2	Z	1
T2_i2_C	T2	C	2

T2_i2_Z	T2	Z	2
T2_i3_C	T2	C	3
T2_i3_Z	T2	Z	3
S2_i1_C	S2	C	1
S2_i1_Z	S2	Z	1
S2_i2_C	S2	C	2
S2_i2_Z	S2	Z	2
S2_i3_C	S2	C	3
S2_i3_Z	S2	Z	3

Design matrix:

~Pop + Pop:Ind + Pop:Cond

Contrasts:

**Contrast**	**Genes**	**Gene set**
PopT1.CondZ	T1 in control vs. T1 in zinc	g (Extended Data Fig. 3)
PopS1.CondZ	S1 in control vs. S1 in zinc	e (Extended Data Fig. 3)
PopT2.CondZ	T2 in control vs. T2 in zinc	h (Extended Data Fig. 3)
PopS2.CondZ	S2 in control vs. S2 in zinc	f (Extended Data Fig. 3)

5.3 **Within-condition comparisons (ecotype level):**

Individual assignments:

**Sample**	**Eco_Cond**
T1_i1_C	TC
T1_i1_Z	TZ
T1_i2_C	TC
T1_i2_Z	TZ
T1_i3_C	TC
T1_i3_Z	TZ
S1_i1_C	SC
S1_i1_Z	SZ
S1_i2_C	SC
S1_i2_Z	SZ
S1_i3_C	SC
S1_i3_Z	SZ
T2_i1_C	TC
T2_i1_Z	TZ
T2_i2_C	TC

T2_i2_Z	TZ
T2_i3_C	TC
T2_i3_Z	TZ
S2_i1_C	SC
S2_i1_Z	SZ
S2_i2_C	SC
S2_i2_Z	SZ
S2_i3_C	SC
S2_i3_Z	SZ

Design matrix:

~Eco_Cond

Contrasts:

**Contrast**	**Genes**	**Gene Set**
SZ vs.TZ	S vs. T in zinc	Substantial |EC|

5.4 Between-condition comparisons (ecotype level)

Individual assignments:

**Sample**	**Ecotype**	**Cond**	**Ind**
T1_i1_C	T	C	1
T1_i1_Z	T	Z	1
T1_i2_C	T	C	2
T1_i2_Z	T	Z	2
T1_i3_C	T	C	3
T1_i3_Z	T	Z	3
S1_i1_C	S	C	1
S1_i1_Z	S	Z	1
S1_i2_C	S	C	2
S1_i2_Z	S	Z	2
S1_i3_C	S	C	3
S1_i3_Z	S	Z	3
T2_i1_C	T	C	4
T2_i1_Z	T	Z	4
T2_i2_C	T	C	5
T2_i2_Z	T	Z	5
T2_i3_C	T	C	6
T2_i3_Z	T	Z	6
S2_i1_C	S	C	4
S2_i1_Z	S	Z	4

S2_i2_C	S	C	5
S2_i2_Z	S	Z	5
S2_i3_C	S	C	6
S2_i3_Z	S	Z	6

Design matrix:

~Ecotype + Ecotype:Ind + Ecotype:Cond

Contrasts:

**Contrast**	**Genes**	**Gene set**
EcotypeS.CondZ	Susceptible individuals control vs. zinc	Substantial |PC|

Supplementary File 1 (File_S1.csv): Overrepresented Gene Ontology (GO) terms for genes upregulated in the zinc in S1 and S2.

For genes upregulated from control to zinc in both S1 and S2 in the same direction, table outlining enriched GO terms. Each row indicates a GO ID with columns indicating: i) the GO_ID; ii) overrepresented p-value; iii) underrepresented p-value; iv) the number of genes in the background set; v) the number of genes in the query set; vi) the GO term; vii) the GO ontology; viii) the over-represented false discovery rate; and ix) the GO term.

Supplementary File 2 (File_S2.csv): **Overrepresented Gene Ontology (GO) terms for genes differentially expressed between S1 and T1, and S2 and T2, in the zinc treatment.**

For genes differentially expressed between T1 and S1, and T2 and S2, in the zinc treatment, in the same direction, table outlining enriched GO terms. Each row indicates a GO ID with columns indicating: i) the GO_ID; ii) overrepresented p-value; iii) underrepresented p-

value; iv) the number of genes in the background set; v) the number of genes in the query set; vi) the GO term; vii) the GO ontology; viii) the overrepresented false discovery rate; and ix) the GO term.

Supplementary File 3 (File_S3.csv): Overrepresented Gene Ontology (GO) terms for derived plasticity (DP) genes with ancestral plasticity. For genes displaying the DP expression pattern (see main text for definition), which also showed differential expression between treatments in S1 and S2, table outlining enriched GO terms. Each row indicates a GO ID with columns indicating: i) the GO_ID; ii) overrepresented p-value; iii) underrepresented p-value; iv) the number of genes in the background set; v) the number of genes in the query set; vi) the GO term; vii) the GO ontology; viii) the overrepresented false discovery rate; and ix) the GO term.

Supplementary File 4 (File_S4.csv): Overrepresented Gene Ontology (GO) terms for derived plasticity (DP) genes without ancestral plasticity. For genes displaying the DP expression pattern (see main text for definition), but which did not show differential expression between treatments in S1 and S2, table outlining enriched GO terms. Each row indicates a GO ID with columns indicating: i) the GO_ID; ii) overrepresented p-value; iii) underrepresented p-value; iv) the number of genes in the background set; v) the number of genes in the query set; vi) the GO term; vii) the GO ontology; viii) the overrepresented false discovery rate; and ix) the GO term.

Supplementary File 5 (File_S5.csv): Overrepresented Gene Ontology (GO) terms for constitutive expression change (CEC) genes. For genes displaying the CEC expression pattern (see main text for definition), table outlining enriched GO terms. Each row indicates a GO ID with columns indicating: i) the GO_ID; ii) overrepresented p-value; iii) underrepresented p-value; iv) the number of genes in the background set; v) the number of genes in the query set; vi) the GO term; vii) the GO ontology; viii) the overrepresented false discovery rate; and ix) the GO term.

Supplementary File 6 (File_S6.csv): Overrepresented Gene Ontology (GO) terms for derived plasticity (DP) genes. For genes displaying the DP expression pattern (see main text for definition), table outlining enriched GO terms. Each row indicates a GO ID with columns indicating: i) the GO_ID; ii) overrepresented p-value; iii) underrepresented p-value; iv) the number of genes in the background set; v) the number of genes in the query set; vi) the GO term; vii) the GO ontology; viii) the overrepresented false discovery rate; and ix) the GO term.

Supplementary File 7 (File_S7.csv): Data for individual RNA samples. For each individual sequenced, table outlining i) the sample ID, ii) the date of extraction, iii) the Plant ID, iv) the population ID, v) RNA concentration measured on a Nanodrop 3000, vi) the 260/280nm absorbance ratio, vii) the 260/230 absorbance ratio, viii) RNA concentration measured on a Qubit, ix) the elution volume (first + second elution), x) the extraction batch, xi) the RNA integrity number (RIN), xii) the number of raw reads, xiii)

the number of clean reads produced per sample, and xiv) whether that sample was used in *de novo* transcriptome construction.

Supplementary File 8 (File_S8.phylip.txt): Phylip format file of sites used to construct the phylogenetic tree in Figure 1A.

Supplementary Table 1: Impact of parametric bootstrapping on response to ancestral plasticity classification.

Classifications of genes showing substantial ancestral plasticity and evolutionary change from i) the entire transcriptome, ii) genes showing derived plasticity DP genes), iii) genes showing constitutive expression changes (CEC) by their evolutionary response to ancestral plasticity; reversion, overshooting or reinforcement. Listed either without parametric bootstrapping or after parametric bootstrapping (the % of genes followed by the number able to be classified).

Gene set	Class	No bootstrapping	Bootstrapping
Entire transcriptome	Reversion	95.2% (12,070)	98.5% (8,952)
	Overshooting	3.7% (465)	0.7% (67)
	Reinforcement	1.1% (144)	0.8% (72)
DP Genes	Reversion	79.6% (90)	91.5% (86)
	Overshooting	16.8% (19)	5.3% (5)
	Reinforcement	3.5% (4)	3.2% (3)
CEC Genes	Reversion	68.4% (154)	74.9% (128)
	Overshooting	3.6% (8)	1.8% (3)
	Reinforcement	28.0% (63)	23.4% (40)

Supplementary Table 2: Classification of evolutionary response to ancestral plasticity in geographic pairs.

Table outlining for genes in i) the entire transcriptome, ii) DP genes, iii) genes with derived plasticity in only one of T1 or T2, iii) CEC genes, iv) CEC genes and v) genes with constitutive expression changes in only one of T1 or T2, evolutionary responses to ancestral plasticity. For each gene set, the percentage of genes that could be classified (that showed substantial ancestral plasticity and evolutionary change; see Methods) and the proportions assigned to overshooting, reversions and reinforcement are given. The values are either calculated based on values of L_o, L_p and L_a calculated from a) T1 and S1 only (i.e. L_p is average of S1 expression in zinc; L_a is expression of T1 in the zinc etc.), b) T2 and S2 only (i.e. L_p is average of S2 expression in zinc; L_a is expression of T2 in the zinc etc.) and c) the combined values (i.e. L_p is average of S1 and S2 expression in zinc; L_a is expression of T1 and T2 in the zinc etc.; see Methods); note that these are the values used in the text unless otherwise stated.

	**T1/S1**	**T2/S2**	**Combined**
% of whole transcriptome classified	36.1%	37.1%	45.3%
Whole transcriptome classifications:<br>Overshooting<br>Reversion<br>Reinforcement	 12.3%<br>83.8%<br>3.9%	 7.7%<br>87.3%<br>5.1%	 3.7%<br>95.2%<br>1.1%
% of DP genes classified	81.0%	81.0%	82.5%
DP gene classifications:<br>Overshooting<br>Reversion<br>Reinforcement	 12.6%<br>82.8%<br>4.5%	 16.2%<br>81.1%%<br>2.7%	 16.8%<br>79.6%<br>3.5%%
% of genes with derived plasticity in only one of T1 or T2 classified	73.1%	71.8%	NA

Genes with derived plasticity in only one of T1 or T2 classifications<br>Overshooting<br>Reversion<br>Reinforcement	 <br>15.8%<br>76.3%<br>7.9%	 <br>11.1%<br>84.2%<br>4.6%	 <br>NA<br>NA<br>NA
% of CEC genes classified	44.0%	42.0%	56.2%
CEC gene classifications:<br>Overshooting<br>Reversion<br>Reinforcement	 5.11%<br>74.4%<br>20.5%	 3.6%<br>79.2%<br>17.3%	 3.6%%<br>68.4%<br>28.0%
% of genes with constitutive expression changes in only one of T1 or T2 classified	37.5%	35.3%	NA
Genes with constitutive expression changes in only one of T1 or T2 classifications<br>Overshooting<br>Reversion<br>Reinforcement	 <br>13.5%<br>74.9%<br>11.6%	 <br>11.2%<br>80.5%<br>8.3%	 <br>NA<br>NA<br>NA

Supplementary Table 3: Evolutionary responses to ancestral plasticity using different criteria for defining "substantial" ancestral plasticity (PC) and evolutionary change (EC). Table outlining evolutionary responses to ancestral plasticity (metrics outlined in table rows), using genes with "substantial" EC and PC defined using each of the three methods outlined in the Supplementary Methods. i) a significance threshold for gene expression changes in susceptible populations between conditions (PC) and between susceptible and tolerant populations in zinc (EC), ii) the magnitude of PC and EC being greater than 20% of the average expression level of the gene in ancestral populations in control conditions (L_o**),** iii) significant differential expression between conditions in both S1 and S2, and between S1 and T1 and S2 and T2 in the zinc, in addition to the criteria outlined in (ii). Method (i) is used in the main text.

**Result**	**Significance threshold for EC and PC (main text; See Methods)**	**|EC| and |PC| > 0.2*Lo**	**|EC| and |PC| > 0.2*Lo, differentially expressed in both susceptible populations between treatments, differentially expressed between both tolerant and sensitive populations in zinc.**
Proportion of whole transcriptome passing "substantial EC and PC" threshold	45.3%	75.6%	29.3%
Whole transcriptome response to ancestral plasticity	Reversion=95.2%, Overshooting=3.7%, Reinforcement=1.1%	Reversion=84.8%, Overshooting=9.5%, Reinforcement=5.7%	Reversion=96.9%, Overshooting=2.5%, Reinforcement=0.6%
Proportion of DP genes passing "substantial EC and PC threshold"	82.5%	86.9%	76.7%
DP gene response to ancestral plasticity	Reversion=79.6%, Overshooting=16.8%, Reinforcement=3.5%	Reversion=79.8%, Overshooting=12.6%, Reinforcement=7.6%	Reversion=81.9%, Overshooting=14.3%, Reinforcement=3.8%
Proportion of CEC genes passing "substantial EC and PC threshold"	56.2%	74.5%	38.0%
CEC gene response to ancestral plasticity	Reversion=68.4%, Overshooting=3.6%, Reinforcement=28.0%	Reversion=63.8%, Overshooting=3.0%, Reinforcement=33.2%	Reversion=85.5%%, Overshooting=0.7%, Reinforcement=13.8%
p-value for CEC genes showing more	i = $<2.2\times10^{-16}$<br>ii = $<2.2\times10^{-16}$	i = $<2.2\times10^{-16}$<br>ii = $<2.2\times10^{-16}$	i = 3.9×10^{-7}<br>ii = 2.2×10^{-16}

reinforcement than i) DP genes and ii) whole transcriptome			
Proportion of genes with CEC expression pattern in only i) T1 or ii) T2 showing reversion.	i = 74.9%<br>ii = 80.5%	i = 77.2%<br>ii = 82.8%	i = 77.2%<br>ii = 82.8%
Proportion of genes with DP expression pattern in only i) T1 or ii) T2 showing reversion	i = 76.3%<br>ii = 84.2%	i = 80.5%<br>ii = 86.1%	i = 80.5%<br>ii = 86.1%
p-value for genes showing ancestral plasticity vs. no ancestral plasticity having different |FC| similarity in T1 vs. T2 in the zinc in i) DP genes and ii) CEC genes	i = 0.18<br>ii = 0.86	i = 0.98<br>ii = 0.88	i = 0.82<br>ii = 0.64
Percent of originally classified genes classified after permutation for i) Whole transcriptome,	i = 71.7%<br>ii = 83.2%<br>iii = 76.0%	i = 46.4%<br>ii = 77.3%<br>iii = 55.0%	i = 82.8%<br>ii = 84.8%<br>iii = 85.5%

ii) DP genes and iii) CEC genes			
Whole transcriptome response to ancestral plasticity after permutations	Reversion=98.5%, Overshooting=0.7%, Reinforcement=0.8%	Reversion = 98.9%, Overshooting = 0.4% Reinforcement = 0.8%	Reversion=99.6%, Overshooting=0.3%, Reinforcement=0.2%
DP gene response to ancestral plasticity after permutations	Reversion=91.5.%, Overshooting=5.3%, Reinforcement=3.2%	Reversion = 93.5%, Overshooting = 4.3%, Reinforcement = 2.1%	Reversion=94.4%, Overshooting=4.4%, Reinforcement=1.1%
CEC gene response to ancestral plasticity after permutations	Reversion=74.9%, Overshooting=1.7%, Reinforcement=23.4%	Reversion = 81.1%, Overshooting = 0.6%, Reinforcement =18.3%	Reversion=91.3%, Overshooting=0.8%, Reinforcement=7.9%

Supplementary Table 4: BUSCO assembly scores for individual and combined transcriptome assemblies. The proportion of BUSCO single copy genes in the from the Eudicot dataset present as i) single copy, ii) duplicated, iii) fragmented and iv) missing in de novo assemblies of 4 individuals (1 per population, outlined in brackets), plus the combined de novo assembly using all the data from these 4 individuals.

	**BD1 (S1)**	**SA6 (S2)**	**GR10 (T1)**	**PP1 (T2)**	**Combined**
Single copy	66.2%	68.4%	71.3%	69.4%	72.8%
Duplicated	2.1%	2.4%	2.7%	2.3%	2.7%
Fragmented	8.5%	8.1%	7.1%	8.5%	8.3%
Missing	23.2%	21.1%	18.9%	19.8%	16.2%

Supplementary Table 5: Evolutionary responses to ancestral plasticity, controlling or not controlling for an effect of the genotypes of paired clones between treatments. Comparison of the method of identifying differentially expressed genes between conditions; i) where effects of genotypes of the paired clones between treatments are controlled for by employing a model of ~Pop + Pop:Ind + Pop:Cond (with contrasts PopS1.CondZ, PopS2.CondZ etc.) and ~Ecotype + Ecotype:Ind + Ecotype:Cond (with contrast EcotypeS.CondZ), as in the main text, or ii) where no effect of genotype is included, by using the models ~Pop_Cond (using contrasts S1C vs. S1Z, T1C vs. T1Z etc.) and ~Eco_Cond (using contrasts SC vs. SZ). For each of these methods, for the i) Entire transcriptome, ii) DP genes and ii) CEC genes, the total number of genes and those classified as undergoing reversion (REV), overshooting (OVER) or reinforcement (RI) are

displayed; as a percentage of the total number of genes showing substantial |EC| and |PC|, with the raw number of genes in brackets.

**Gene set**	**Metric**	**Controlling for genotype (main text)**	**Not controlling for genotype**
Entire transcriptome	REV	95.2% (12,070)	95.5% (11,169)
	OVER	3.7% (465)	4.0% (466)
	RI	1.1% (144)	0.6% (65)
DP Genes	Number of genes	137	46
	REV	79.6% (90)	71.1% (27)
	OVER	16.8% (19)	23.7% (9)
	RI	3.5% (4)	5.3% (2)
CEC Genes	Number of genes	400	400
	REV	68.4% (154)	73.9% (125)
	OVER	3.6% (8)	4.7% (8)
	RI	28.0% (63)	21.3% (36)

SI References

1. Papadopulos, A. S. T. *et al*. Rapid parallel adaptation to anthropogenic heavy metal Pollution. *Molecular Biology and Evolution* 38, 3724–3736 (2021).
2. Andrews, S. A. FATSQC, a quality control tool for high throughput sequence data. 2010. (2015).
3. Patel, R. K. & Jain, M. NGS QC Toolkit: A toolkit for quality control of next generation sequencing data. *PLoS ONE* 7, e30619 (2012).
4. Bolger, A. M., Lohse, M. & Usadel, B. Trimmomatic: a flexible trimmer for illumina sequence data. *Bioinformatics* 30, 2114–2120 (2014).

5. Haas, B. J. *et al*. De novo transcript sequence reconstruction from RNA-seq using the Trinity platform for reference generation and analysis. *Nature Protocols* 8, 1494–1512 (2013).
6. Bray, N. L., Pimentel, H., Melsted, P. & Pachter, L. Near-optimal probabilistic RNA-seq quantification. *Nature Biotechnology* 34, 525–527 (2016).
7. Williams, A. M., Itgen, M. W., Broz, A. K., Carter, O. G. & Sloan, D. B. Long-read transcriptome and other genomic resources for the angiosperm *Silene noctiflora*. *G3: Genes, Genomes, Genetics* 11, (2021).
8. Seppey, M., Manni, M. & Zdobnov, E. M. BUSCO: Assessing genome assembly and annotation completeness. in Methods in Molecular Biology vol. 1962 227–245 (Humana Press Inc., 2019).
9. Kent, W. J. BLAT---The BLAST-Like Alignment Tool. *Genome Research* 12, 656–664 (2002).
10. Bateman, A. UniProt: A worldwide hub of protein knowledge. *Nucleic Acids Research* 47, D506–D515 (2019).
11. Altschul, S. F., Gish, W., Miller, W., Myers, E. W. & Lipman, D. J. Basic local alignment search tool. *Journal of Molecular Biology* 215, 403–410 (1990).
12. Stranger, B. E. *et al*. Relative impact of nucleotide and copy number variation on gene expression phenotypes. *Science* 315, 848–853 (2007).
13. Craciun, A. R. *et al*. Variation in HMA4 gene copy number and expression among *Noccaea caerulescens* populations presenting different levels of Cd tolerance and accumulation. *Journal of Experimental Botany* 63, 4179–4189 (2012).
14. Finn, R. D. et al. Pfam: The protein families database. *Nucleic Acids Research* vol. 42 D222 (2014).
15. Mistry, J., Finn, R. D., Eddy, S. R., Bateman, A. & Punta, M. Challenges in homology search: HMMER3 and convergent evolution of coiled-coil regions. *Nucleic Acids Research* 41, e121–e121 (2013).
16. Bryant, D. M. et al. A tissue-mapped axolotl *de novo* transcriptome enables identification of limb regeneration factors. *Cell Reports* 18, 762–776 (2017).
17. Ghalambor, C. K. *et al*. Non-adaptive plasticity potentiates rapid adaptive evolution of gene expression in nature. *Nature* 525, 372–375 (2015).
18. Ho, W. C. & Zhang, J. Genetic gene expression changes during environmental adaptations tend to reverse plastic changes even after the correction for statistical nonindependence. *Molecular Biology and Evolution* 36, 604–612 (2019).
19. Kim, D., Paggi, J. M., Park, C., Bennett, C. & Salzberg, S. L. Graph-based genome alignment and genotyping with HISAT2 and HISAT-genotype. *Nature Biotechnology* 37, 907–915 (2019).
20. Li, H. *et al*. The Sequence Alignment/Map format and SAMtools. *Bioinformatics* 25, 2078–2079 (2009).
21. Li, H. A statistical framework for SNP calling, mutation discovery, association mapping and population genetical parameter estimation from sequencing data. *Bioinformatics* 27, 2987–2993 (2011).
22. Lee, T. H., Guo, H., Wang, X., Kim, C. & Paterson, A. H. SNPhylo: A pipeline to construct a phylogenetic tree from huge SNP data. *BMC Genomics* 15, 162 (2014).
