# Supplementary material for: Genetic assimilation of ancestral plasticity during parallel adaptation to zinc contamination in Silene uniflora
Source: Nat Ecol Evol. 2023 Jan 26;7(3):414–23. doi: 10.1038/s41559-022-01975-w (PMC9998271; doi:10.1038/s41559-022-01975-w)
Supplement: Supplementary file 2 — Reporting Summary [file 41559_2022_1975_MOESM2_ESM.pdf]

nature portfolio

Corresponding author(s): Alexander S.T. Papadopulos

Last updated by author(s): Nov 23, 2022

Reporting Summary

Nature Portfolio wishes to improve the reproducibility of the work that we publish. This form provides structure for consistency and transparency in reporting. For further information on Nature Portfolio policies, see our Editorial Policies and the Editorial Policy Checklist.

Statistics

For all statistical analyses, confirm that the following items are present in the figure legend, table legend, main text, or Methods section.

n/a	Confirmed	
☐	☒	The exact sample size (*n*) for each experimental group/condition, given as a discrete number and unit of measurement
☐	☒	A statement on whether measurements were taken from distinct samples or whether the same sample was measured repeatedly
☐	☒	The statistical test(s) used AND whether they are one- or two-sided<br>*Only common tests should be described solely by name; describe more complex techniques in the Methods section.*
☒	☐	A description of all covariates tested
☐	☒	A description of any assumptions or corrections, such as tests of normality and adjustment for multiple comparisons
☐	☒	A full description of the statistical parameters including central tendency (e.g. means) or other basic estimates (e.g. regression coefficient) AND variation (e.g. standard deviation) or associated estimates of uncertainty (e.g. confidence intervals)
☐	☒	For null hypothesis testing, the test statistic (e.g. *F*, *t*, *r*) with confidence intervals, effect sizes, degrees of freedom and *P* value noted<br>*Give P values as exact values whenever suitable.*
☒	☐	For Bayesian analysis, information on the choice of priors and Markov chain Monte Carlo settings
☒	☐	For hierarchical and complex designs, identification of the appropriate level for tests and full reporting of outcomes
☒	☐	Estimates of effect sizes (e.g. Cohen's *d*, Pearson's *r*), indicating how they were calculated

Our web collection on statistics for biologists contains articles on many of the points above.

Software and code

Policy information about availability of computer code

Data collection	NA
Data analysis	FastQC v0.11.8 (read QC), NGSQCToolkit v2.3.3 (read trimming), Trimmomatic v0.39 (read trimming), Trinity v2.10.0 (transcriptome assembly), Transdecoder v5.5.0 (predicting open reading frames), kallisto v0.46 (mapping reads to transcriptome for gene expression estimation), BLAT v35 (mapping transcripts to Silene latifolia genome), BLASTP v.2.10.0 (mapping transcripts to SwissProt/UniProt atabase), BUSCO v4.0.5 (assessing transcriptome completeness), hmmer v3.3 (transcriptome annotation), trinotate v3.2.1 (transcriptome annotation), HISAT2 v2.2.1 (mapping reads to transcriptome for genotyping), samtools v1.7 (removing reads with low mapping quality), SNPhylo v2014071 (phylogenetic tree construction), DESeq2 v1.2.6 (differential gene expression), tximport v1.14.2 (differential gene expression), R v 3.3.6 (statistical analysis), R v3.63, DESEq2_script_formal_v13.R (https//github.com/danielwood1992/Silene_RNASeq - custom R script for analysis), wesanderson 0.3.6 (R package, color palettes), RcolorBrewer v.1.1-2 (R package, color palettes), ggsci v2.9 (R package, color palettes), reshape2 v.1.4.4 (R package, statistical analysis), VennDiagram v1.71 (R package, plotting), ggnewscale v0.4.5 (R package, plotting), cowplot v1.1.1 (R package, plotting), ggplot2 v3.3.5 (R package, plotting), grdevices v3.6.3 (R package, plotting), ape v1.4.4 (R package, phylogeny plotting), ggtree v3.14 (R package, phylogeny plotting), phytools v0.7.90 (R package, phylogeny plotting), bcftools v1.10.2 (SNP calling).

For manuscripts utilizing custom algorithms or software that are central to the research but not yet described in published literature, software must be made available to editors and reviewers. We strongly encourage code deposition in a community repository (e.g. GitHub). See the Nature Portfolio guidelines for submitting code & software for further information.

Data

Policy information about availability of data

All manuscripts must include a data availability statement. This statement should provide the following information, where applicable:

- Accession codes, unique identifiers, or web links for publicly available datasets
- A description of any restrictions on data availability
- For clinical datasets or third party data, please ensure that the statement adheres to our policy

RNA-seq data is deposited on the NCBI databases under Bioproject PRJNA706929.

Field-specific reporting

Please select the one below that is the best fit for your research. If you are not sure, read the appropriate sections before making your selection.

☐ Life sciences ☐ Behavioural & social sciences ☒ Ecological, evolutionary & environmental sciences

For a reference copy of the document with all sections, see nature.com/documents/nr-reporting-summary-flat.pdf

Ecological, evolutionary & environmental sciences study design

All studies must disclose on these points even when the disclosure is negative.

Study description	This study aimed to identify whether zinc-tolerant populations of Silene uniflora have evolved parallel gene expression changes, and whether these gene expression changes were facilitated by ancestrally plastic responses to zinc. For each of 2 mine populations, and their nearest zinc-sensitive coastal populations (as identified in Papadopulos et al. 2021), 3 individuals were grown from wild collected seed and 6 cloned cuttings were propagated per individual; half of which were exposed to a neutral hydroponic solution, and half to a zinc-contaminated solution. Root tissue of cloned cuttings from a single individual were pooled in each treatment, and RNA extracted and sequenced. Gene expression profiles were calculated and compared between populations to identify the extent of shared gene expression changes in mine populations, and the extent to which these were facilitated by ancestral plastic responses to zinc (determined from the coastal zinc sensitive populations).
Research sample	Seeds were sampled previously at two mine sites and coastal sites where S. uniflora was known to occur. The nearest coastal populations to mine populations were identified using the BSBI database.
Sampling strategy	For the four sites, metal tolerance has previously been conducted and the evolutionary relationships of the populations established using reduced representation sequencing and phylogenetic/population genetic methods. At each site, we collected seeds from a minimum of twelve individuals, which were dried and stored separately with silica gel. Each individual grown from the wild seed was collected from a different individual at each site. Sample sizes were chosen to provide an accurate estimate gene expression changes between populations and treatments.
Data collection	Plants were grown by JAH and DPW. Hydroponics experiments and RNA extraction was performed by DPW, library preparation and sequencing were performed externally by the Beijing Genomics Institute.
Timing and spatial scale	Seeds were collected between 2015-2017. Plants were grown from seed in October 2019; 10 weeks later cuttings were taken and grown for 5.5 weeks (timings allowed plants/cuttings to grow to sufficient size for experimentation). Rooted cuttings were transferred to 6 48x39x20cm 8L hydroponics tanks in a greenhouse in a neutral solution for 1 week then exposed either to refreshed neutral solution or zinc contaminated solution for 8 days before root tissue was harvested.
Data exclusions	No samples were excluded.
Reproducibility	3 replicate hydroponic tanks per treatment each contained one cutting per individual; these were later pooled and analysed together. The whole experiment was not repeated.
Randomization	Cuttings of approximately equal size were distributed evenly between replicate hydroponics tanks, with each individual tank having the same proportions of cuttings from each population. Cuttings were randomly distributed within each tank.
Blinding	Blinding was not relevant to the study; data generation did not involve subjective human judgements.

Did the study involve field work? ☒ Yes ☐ No

Field work, collection and transport

Field conditions	Wild seed samples were collected on dry days.
Location	T1 - Grogwynion, Wales 52.331608 -3.887207<br>S1 - Aberystwyth, Wales 52.394825 -4.093914<br>T2 - Priddy Pools, England 51.256935 -2.650950

	S2 - Brean Down, England 51.323284 -3.016996
Access & import/export	Permission to sample at sites was issued by: Natural Resources Wales for the Welsh sites; Natural England for Priddy Pools; the National trust for Brean Down.
Disturbance	No disturbance was caused.

Reporting for specific materials, systems and methods

We require information from authors about some types of materials, experimental systems and methods used in many studies. Here, indicate whether each material, system or method listed is relevant to your study. If you are not sure if a list item applies to your research, read the appropriate section before selecting a response.

Materials & experimental systems

n/a	Involved in the study	
☒	☐	Antibodies
☒	☐	Eukaryotic cell lines
☒	☐	Palaeontology and archaeology
☐	☒	Animals and other organisms
☒	☐	Human research participants
☒	☐	Clinical data
☒	☐	Dual use research of concern

Methods

n/a	Involved in the study	
☒	☐	ChIP-seq
☒	☐	Flow cytometry
☒	☐	MRI-based neuroimaging

Animals and other organisms

Policy information about studies involving animals; ARRIVE guidelines recommended for reporting animal research

Laboratory animals	NA
Wild animals	NA
Field-collected samples	NA
Ethics oversight	No ethical oversight was required as research was on plants.

Note that full information on the approval of the study protocol must also be provided in the manuscript.
